# Effect of Flaxseed Press Cake (*Linum usitatissimum* L.) Incorporation on the Polyphenol Content, Antioxidant Potential, and Quality of Partially Fat-Replaced Cookies

**DOI:** 10.3390/molecules31203752

**Published:** 2026-10-19

**Authors:** Marek Kruczek, Dorota Gumul, Joanna Oracz, Natalia Marriott-Rybka, Agata Kusz

**Affiliations:** 1 Department of Carbohydrate Technology and Cereal Processing, Faculty of Food Technology, University of Agriculture in Krakow, 122 Balicka Street, 30-149 Krakow, Poland; natalkamarriott@gmail.com (N.M.-R.); agata.k522@wp.pl (A.K.); 2 Institute of Food Technology and Analysis, Faculty of Biotechnology and Food Sciences, Lodz University of Technology, Bohdana Stefanowskiego 2/22, 90-537 Lodz, Poland; joanna.oracz@p.lodz.pl

**Keywords:** antioxidant activity, bioactive compounds, cookies, flaxseed press cake, polyphenols, quality

## Abstract

Current trends in food technology include the development of reduced-fat snacks and the use of food industry by-products. This study aimed to develop a formulation for partially fat-replaced cookies enriched with flaxseed press cake (FPS). The study was conducted in three stages: (I) determination of the level of margarine replacement with maltodextrin (5–25%) to identify the most favorable level, (II) comparison of maltodextrins obtained from light-fleshed and purple-fleshed potato starch, and (III) evaluation of the effects of partial wheat flour replacement with FPS (10–30%) on the physicochemical, sensory, and antioxidant properties of the cookies. Replacing 10% of the margarine with maltodextrin with a dextrose equivalent (DE) of 17 provided the most favorable combination of physical and sensory properties among the tested levels. The incorporation of FPS resulted in a dose-dependent increase in total polyphenol content, reaching a value approximately 13.5 times higher than that of the control sample. In vitro antioxidant capacity increased by up to approximately 4.6-fold in the ABTS assay and 4.9-fold in the DPPH assay. HPLC analysis identified eight phenolic acids. Their total content increased from 2.90 mg/100 g DM in the control sample to 17.01 mg/100 g DM in B3J and 19.11 mg/100 g DM in B3F samples, containing 30% FPS and maltodextrin produced using light-fleshed and purple-fleshed potato starch, respectively. Gallic acid was the predominant phenolic acid in the enriched samples, reaching 12.47–13.82 mg/100 g DM. Samples containing maltodextrin obtained from purple-fleshed potato starch (BXF) generally had a higher total content of the identified phenolic compounds than the corresponding samples containing maltodextrin obtained from light-fleshed potato starch (BXJ). Increasing the proportion of FPS to 20–30% increased cookie hardness by up to approximately 3.1-fold and reduced the sensory scores. The most favorable balance between bioactive compound content and product quality was obtained for samples B1J and B1F, which contained 10% FPS. These findings support the use of FPS in the development of partially fat-replaced cookies with enhanced in vitro antioxidant capacity, in line with the principles of the circular economy.

## 1. Introduction

The confectionery industry is one of the most rapidly developing sectors of the food industry in Poland. Poland’s accession to the European Union contributed substantially to the accelerated development of the food industry, including the confectionery sector [1]. According to NielsenIQ, the value of the Polish confectionery market between October 2011 and September 2012 was estimated at 8.2 billion Polish zloty (PLN), while the production volume during this period was approximately 700,000 tonnes [2]. The value of confectionery sales also increased by 6.1% during the same period [3]. Between July 2022 and June 2023, the Polish confectionery market was valued at PLN 15.3 billion, representing an increase of 86.59% compared with the period from October 2011 to September 2012 [4]. Baked confectionery products constitute a diverse food category that can be classified according to shelf life, raw material composition, production technology, and type of dough [5]. In recent years, cookies have become increasingly popular snacks because of their convenience, consumer acceptance, and shelf stability [6,7]. However, these products are often relatively high in fat and sugar, which may be a concern for consumers seeking lower-calorie alternatives. Growing consumer expectations regarding the nutritional and health-related properties of cookies encourage food manufacturers to use healthier substitutes and enrich their products with ingredients that may provide health benefits [8].

Growing consumer awareness of the role of a healthy diet in preventing diet-related chronic diseases has increased interest in alternative approaches to food production. Particular attention has been paid to the development of “light” products with reduced fat and sugar contents, as well as products enriched with bioactive ingredients [9]. Fat replacement presents several technological challenges because fat contributes substantially to the quality and sensory properties of food products. Reducing the fat content of low-moisture, high-fat foods can considerably affect their flavor, appearance, aroma, and texture, thereby altering consumer perception [6]. Maltodextrins can be used as fat replacers primarily because of their ability to form gel-like structures that reproduce the creamy and moist texture normally imparted by fat. Their water-binding properties and ability to form viscous solutions help maintain the structure and volume of baked products while reducing their caloric content [10]. Partial replacement of fat with maltodextrin can reduce the fat content by more than 10% without significantly affecting the sensory acceptability of cookies [11].

Flaxseed (*Linum usitatissimum* L.), a member of the Linaceae family, is an annual crop of considerable economic importance. It is grown mainly in temperate regions of Europe, Asia, and North Africa. Its cultivation in the Middle East dates back to approximately 9000 BCE, making it one of the oldest crops domesticated by humans [12,13,14]. Flaxseed oil is one of the main products obtained from oilseed flax processing and is used in both food production and as a feedstock for biofuels [15,16]. Flaxseed has a high nutritional value and is a rich source of protein, energy, omega-3 polyunsaturated fatty acids, particularly α-linolenic acid, lignans, polyphenols, dietary fiber, vitamins, and minerals. Polyphenols are concentrated mainly in the seed coat, where they occur as esters containing 3-hydroxy-3-methylglutaric acid (HMG) residues and other compounds. These compounds can be released through the hydrolysis of ester bonds, potentially improving their bioavailability [17]. Phenolic acids present in flaxseed, particularly caffeic, ferulic, and *p*-coumaric acids, exhibit strong antioxidant properties. Their total content in flaxseed ranges from 8 to 10 g/kg DM, of which approximately 5 g/kg occurs in esterified form [18,19]. The flavonoid content ranges from 35 to 71 mg/100 g, corresponding to 0.35–0.71 g/kg, depending on the cultivar and growing conditions [18,20]. Flaxseed contains both C- and O-glycosylflavones, together with common cinnamic acids synthesized during the early stages of plant development [21]. It also contains lignin, a complex phenolic polymer with antioxidant and anti-inflammatory properties that performs a structural function in plant cells.

**Figure 1:**
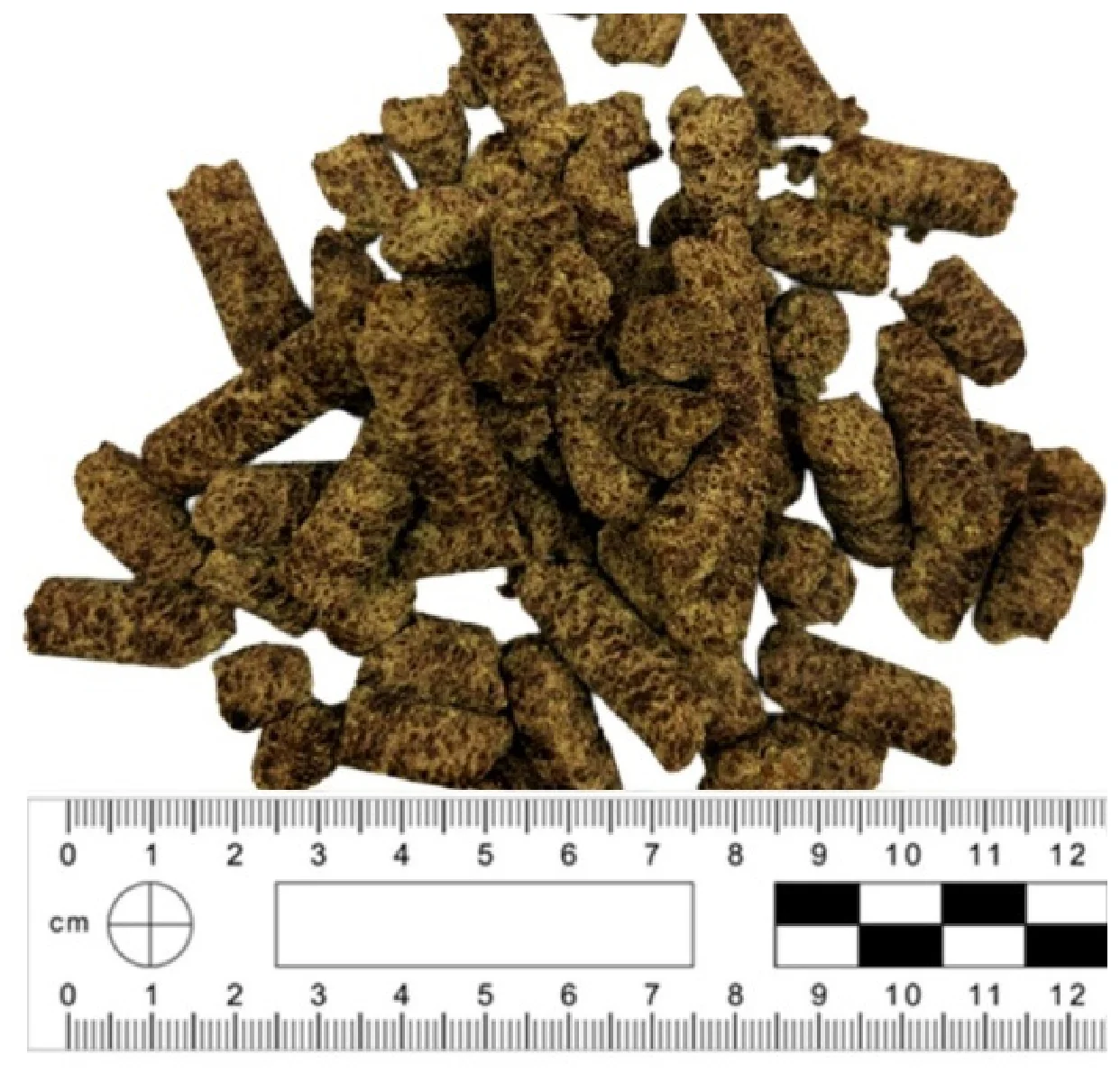
FPS (photograph taken by the authors).

FPS is a by-product of oil pressing (Figure 1). It can be used directly without prior processing or as a raw material for manufacturing other products. It is currently used mainly as animal feed, although it can also provide valuable bioactive compounds in the human diet. FPS contains 7–20% fat, depending on the pressing method, approximately 30–35% protein, and is a concentrated source of lignans. Its phenolic acid content ranges from 1.3 to 2.2 mg/g [22,23,24,25]. FPS is also rich in dietary fiber, which has cholesterol-lowering properties and can reduce intestinal glucose absorption [26].

FPS used as a component of food products is a valuable raw material for the production of protein isolates and hydrolysates with specific functional properties that can be used as functional food ingredients [25,26,27,28]. Nowicka et al. used FPS as a component of chokeberry puree, whereas Ogunronbi et al. [29]. used press cake obtained from dark-brown flaxseed as a flour replacer in bread production [29,30]. Because of its potential health-promoting properties and the growing global interest in the valorization of food-processing residues, FPS is regarded as a valuable ingredient for the food industry that may provide both health and environmental benefits.

The first objective of this study was to determine the effect of the level of fat replacement with maltodextrin and to improve the quality of the cookies. The second objective was to evaluate the effects of different levels of FPS incorporation on total polyphenol and flavonoid contents, the concentrations of individual phenolic compounds determined by HPLC, and the antioxidant activity of partially fat-replaced cookies. The study also included an evaluation of the physical properties of the cookies, including specific volume, color, and mechanical parameters (hardness and cutting energy), as well as the sensory properties of the final products.

## 2. Results and Discussion

### 2.1. Effect of the Level of Margarine Replacement with Maltodextrin on Cookie Quality

Replacing fat in cookies presents a technological challenge because of its important role in determining product flavor and texture. Therefore, the first stage of the study aimed to identify the most favorable level of margarine replacement with maltodextrin within the range of 5–25% based on the evaluated physical and sensory properties. The effects of the margarine replacement level on hardness, cutting energy, specific volume, color, and overall sensory acceptability were evaluated.

Hardness and cutting energy are important quality parameters of confectionery products that directly influence consumer acceptance and the structural stability of the product during storage [31]. In cookies, these properties depend on interactions among the formulation ingredients, including sugar content, fat type, and fatty acid profile, as well as on processing parameters and thermal treatment conditions. Texture analysis of the Stage I cookies showed that partial replacement of margarine with maltodextrin significantly modified their mechanical properties (Table 1). Samples containing 5% and 10% margarine replacement (A1 and A2, respectively) exhibited hardness values that were 30% and 18% lower than that of the control sample. Sample A3_15% showed the greatest similarity to the control, with hardness lower by only 0.4%. Further increases in the margarine replacement level to 20% and 25% increased hardness by 10% and 25%, respectively, compared with the control sample.

Maltodextrin acts as a fat replacer because of its ability to form gel-like structures that reproduce the texture provided by margarine [10]. At lower margarine replacement levels of 5–10%, its strong hygroscopic properties may reduce the availability of water required for the complete development of the gluten network, resulting in a softer and more delicate cookie structure. Qu et al. [32] suggested that, at these levels, maltodextrin may act as a stabilizing filler within the protein matrix and promote changes in protein secondary structure toward β-sheet formation, thereby contributing to the development of a dense and uniform structure.

At margarine replacement levels above 15%, however, the biopolymer matrix becomes more rigid. In these formulations, the proportion of fat is insufficient to effectively separate the flour particles and provide the desired crispness through the shortening effect. Consequently, stronger interchain interactions and denser structural packing contribute to the increased hardness of the cookies [33]. This effect may be further intensified by the phase separation described by Qu et al. [32]. Excess maltodextrin competes with proteins for water, disrupting the continuous gel network and increasing matrix rigidity through the formation of aggregated polysaccharide particles.

**Figure 2:**
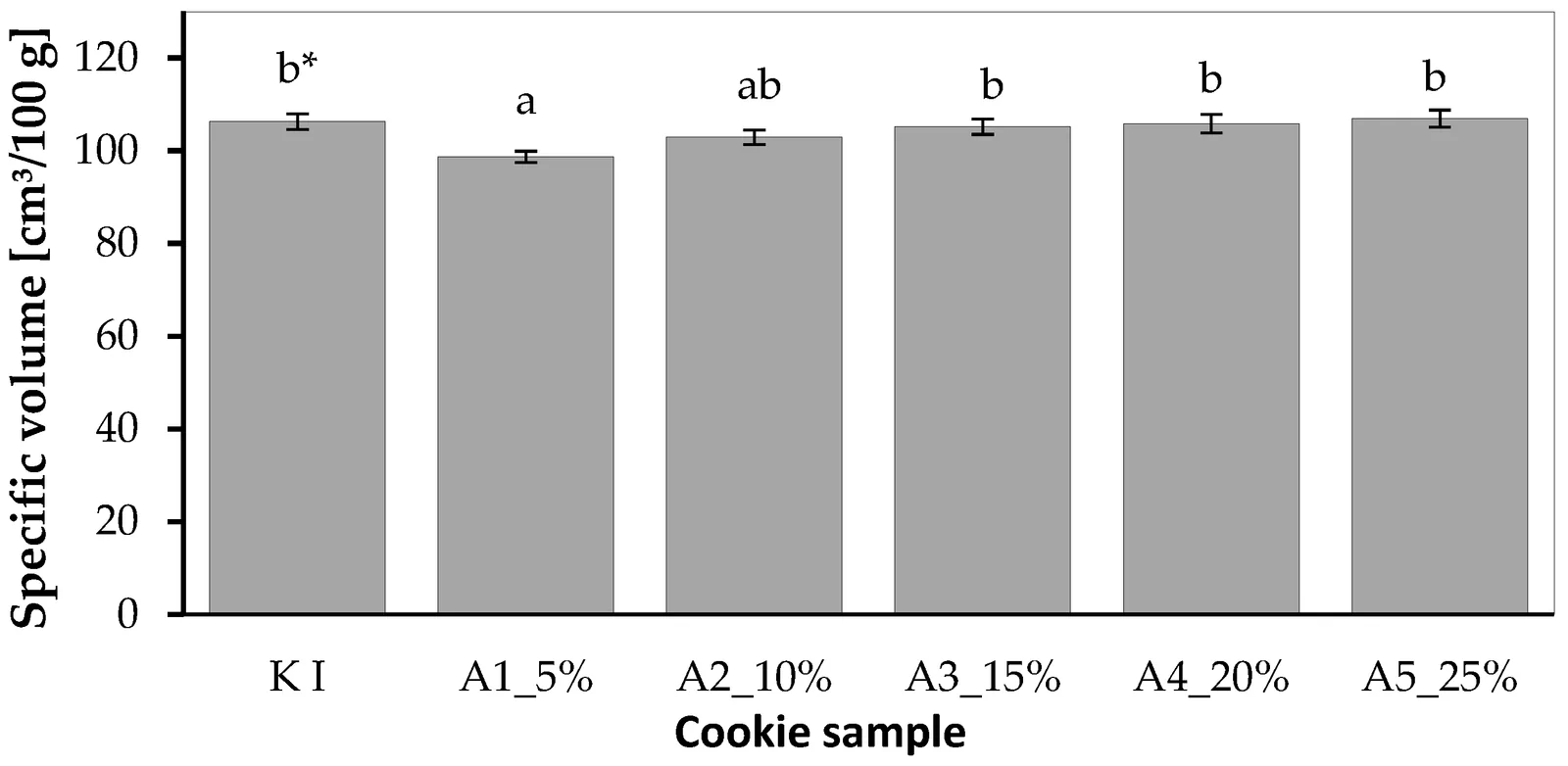
Specific volume [cm^3^/100 g] of the cookie samples analyzed in Stage I. *—Values are presented as mean ± SD (*n* = 5). Different letters above the bars indicate statistically significant differences according to Tukey’s test (*p* < 0.05).

Specific volume is an important quality parameter that reflects both the processing performance and the internal structure of cookies. Replacing 5% and 10% of the margarine with maltodextrin resulted in the greatest reductions in specific volume, amounting to 7% and 3%, respectively, compared with the control sample. Cookies in which 15%, 20%, or 25% of the margarine was replaced with maltodextrin had specific volumes most similar to that of the control sample (Figure 2). The higher maltodextrin replacement levels may have increased dough viscosity and modified its rheological properties, resulting in greater expansion during baking.

**Figure 3:**
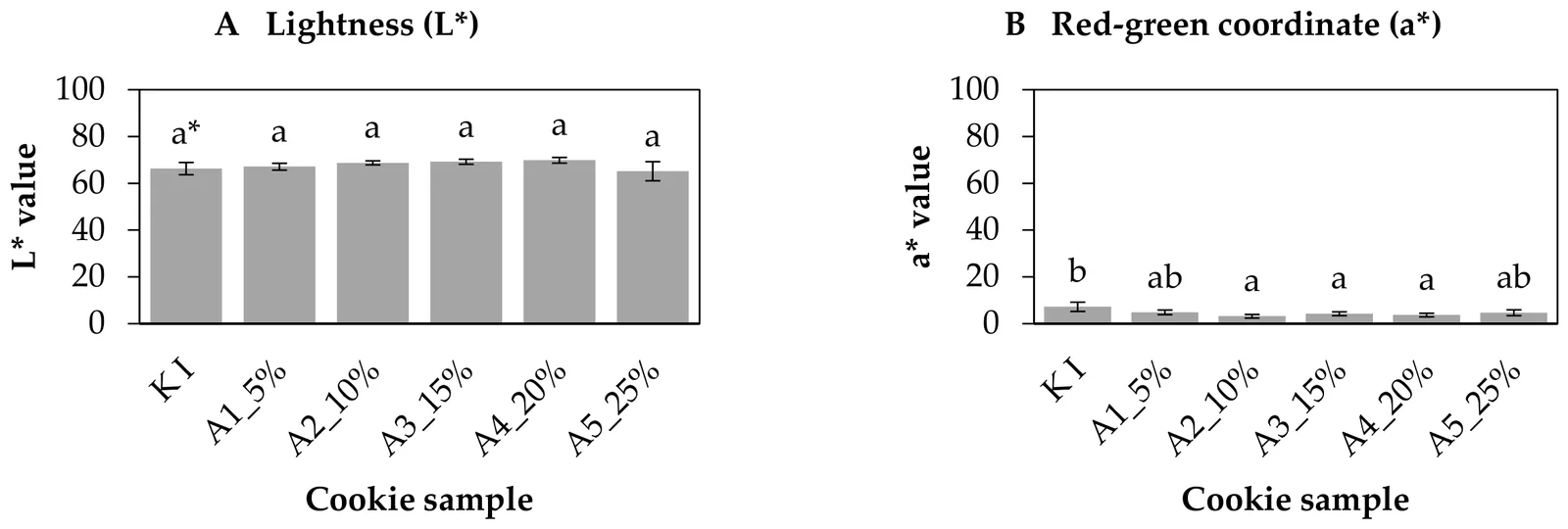

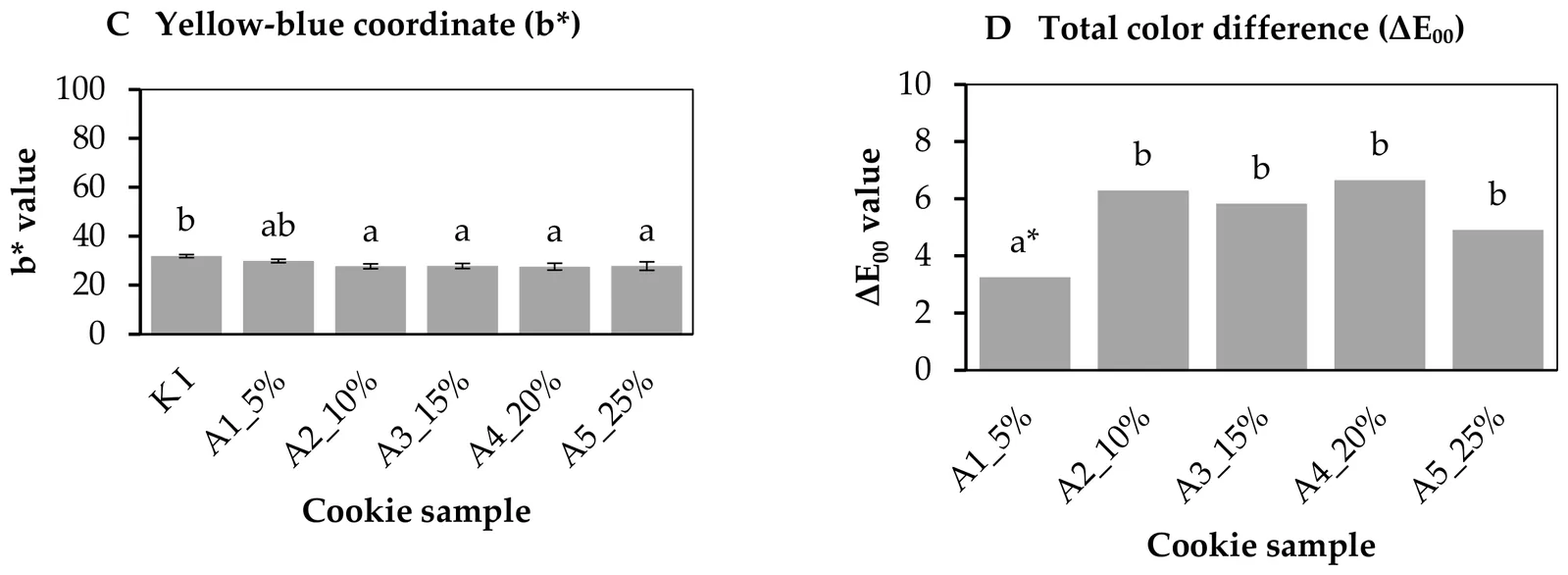
Color parameters of the cookie samples analyzed in Stage I. (**A**–**C**) Values of the L*, a*, and b* color parameters. (**D**) Total color difference (Δ*E*_00_) calculated relative to the control sample. *—values are presented as mean ± standard deviation (*n* = 10). Different letters above the bars indicate statistically significant differences among samples according to Tukey’s test (*p* ≤ 0.05).

The use of maltodextrin as a partial margarine replacer in the cookie formulation significantly affected the color parameters. Samples with 10–20% margarine replacement by maltodextrin (A2–A4) showed a clear increase in lightness, as indicated by their higher L* values, which ranged from 68.75 ± 0.85 to 69.89 ± 1.21, compared with 66.29 ± 1.64 for the control sample. This increase was accompanied by lower a* and b* values, indicating reductions in the red and yellow color components, respectively. As a result, the cookies had a visually lighter and less saturated color. The total color differences (Δ*E*_00_) calculated for A2, A3, and A4 exceeded 5.0 and amounted to 6.29 ± 0.52, 5.83 ± 0.69, and 6.65 ± 1.00, respectively. According to the adopted interpretation scale, these values represent large and readily perceptible color differences. A different trend was observed for A5, in which 25% of the margarine was replaced with maltodextrin. This sample had the lowest lightness among all the analyzed variants (L* = 65.19 ± 1.09; Figure 3).

The observed changes in lightness may be explained by two competing mechanisms occurring within the cookie matrix during thermal processing. At moderate margarine replacement levels of up to 20%, the predominant mechanism is the dilution of the natural pigments present in margarine, such as carotenoids, by white, highly purified maltodextrin. The high intrinsic lightness of maltodextrin, combined with its ability to modify the structure of the matrix and the way in which light is scattered, results in visually lighter products [34,35]. At the highest replacement level of 25%, however, the intensification of the Maillard reaction and nonenzymatic browning becomes the main factor determining product color [36]. As products of starch hydrolysis, maltodextrins contain reducing end groups that, in the presence of flour proteins and at the high baking temperature of 210 °C, promote the formation of dark-colored compounds known as melanoidins [34,37].

**Figure 4:**
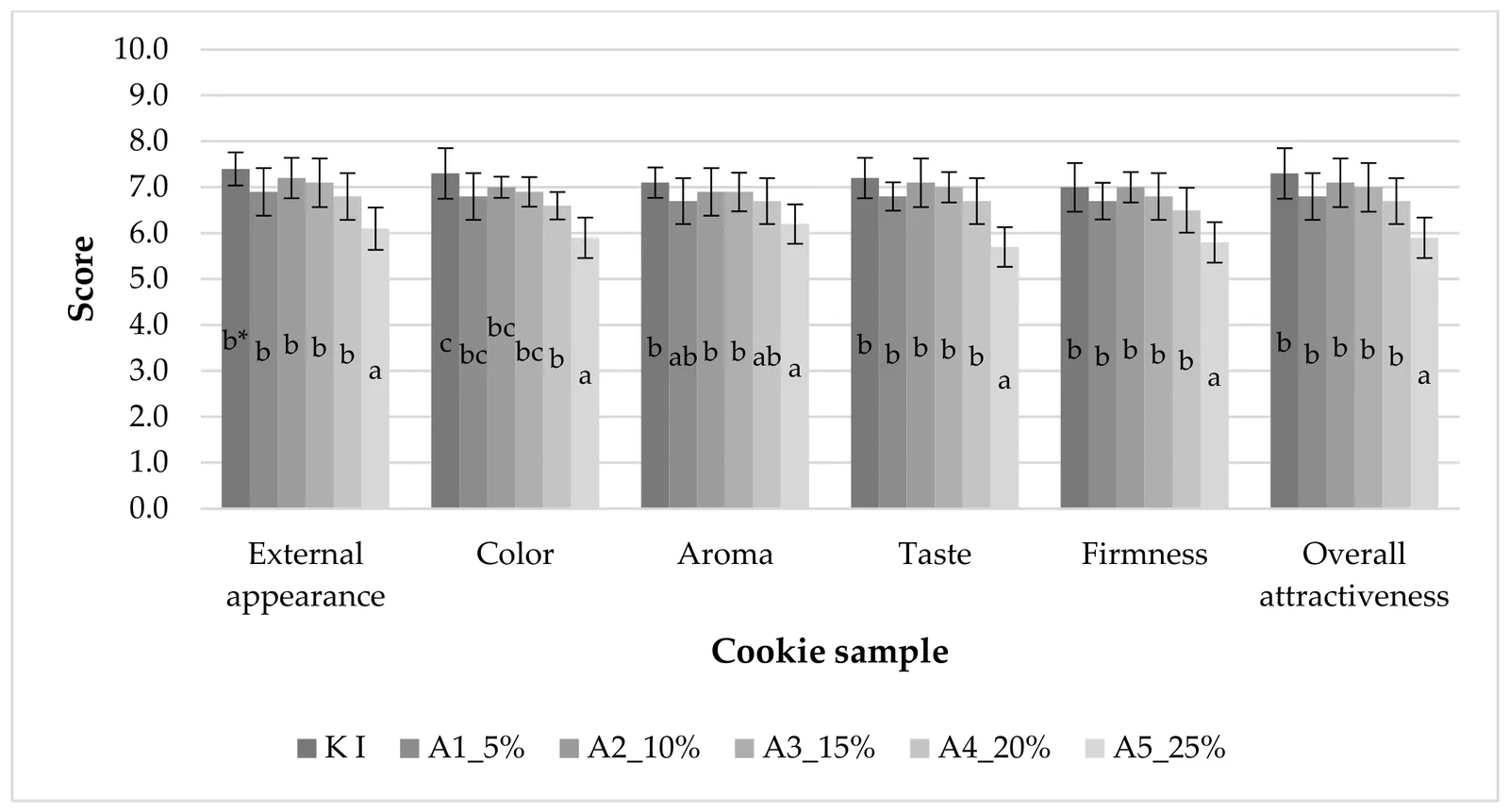
Consumer evaluation of the Stage I cookies with different levels of margarine replacement by maltodextrin). *—Values are presented as mean ± SD (*n* = 14). Different letters indicate statistically significant differences according to Tukey’s test (*p* < 0.05).

The control sample (K I) received the highest overall sensory acceptability score of 7.3 points. Among the variants containing maltodextrin, A2_10% received the highest score of 7.1 points, with particularly favorable ratings for appearance, flavor, color, and hardness. Sample A3_15% received a similar overall score of 7.0 points. Sample A5_25% received the lowest score of 5.9 points, primarily because of its lower flavor rating (Figure 4).

Based on a comparative analysis of the different maltodextrin replacement levels, sample A2_10%, in which 10% of the margarine was replaced with maltodextrin, showed the most favorable combination of physical and sensory properties. Despite its lower hardness and specific volume, this sample had the highest lightness and received the highest sensory acceptability score among the maltodextrin-containing variants. Therefore, a 10% margarine replacement level was selected as the most favorable among the tested levels and was used in the subsequent stages of the study.

### 2.2. Effect of Maltodextrin Source on Cookie Quality

In Stage II, three types of cookies were prepared: control cookies, cookies containing maltodextrin obtained from light-fleshed potato starch, and cookies containing maltodextrin obtained from purple-fleshed potato starch. Texture, color, and sensory analyses were performed to determine whether the source of maltodextrin affected cookie quality (Figure 4).

Texture analysis of the Stage II cookies showed that samples containing maltodextrin obtained from light-fleshed and purple-fleshed potato starch had hardness values that were 21% and 29% lower, respectively, than that of the control sample (Table 2). Cutting energy, which represents the energy required to cut a sample and provides a measure of the structural resistance of cookies to mechanical force, differed significantly from the control only in the sample containing maltodextrin obtained from purple-fleshed potato starch. In this sample, cutting energy increased by approximately 20% compared with the control (Table 2).

**Figure 5:**
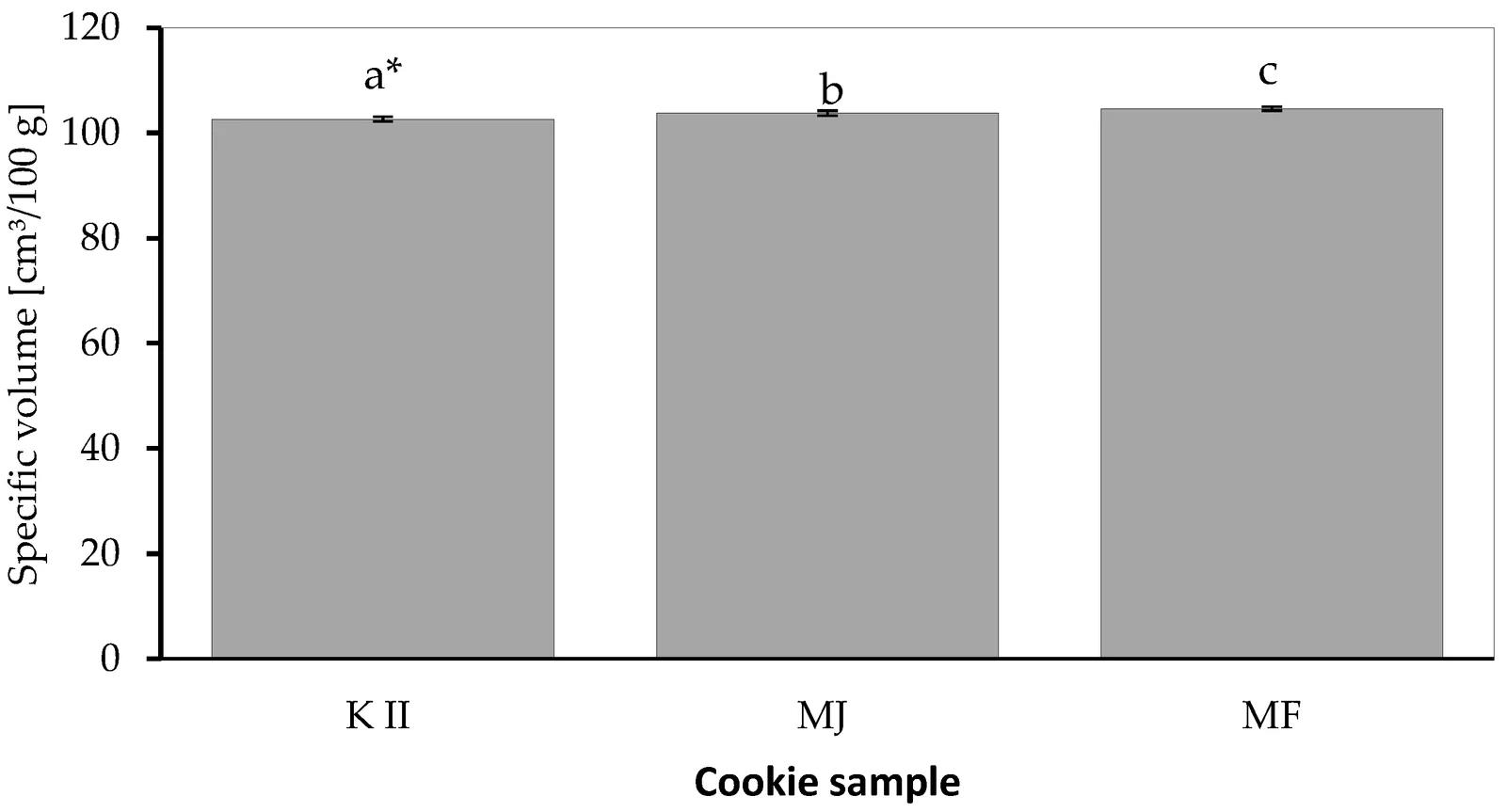
Specific volume [cm*^3^*/100 g] of the cookie samples analyzed in Stage II. *—Values are presented as mean ± SD (*n* = 5). Different letters indicate statistically significant differences according to Tukey’s test (*p* < 0.05).

Specific volume increased slightly relative to the control in both MJ and MF, by 1% and 2%, respectively. Although these differences were statistically significant, their magnitude was unlikely to be perceptible to consumers (Figure 5).

**Figure 6:**
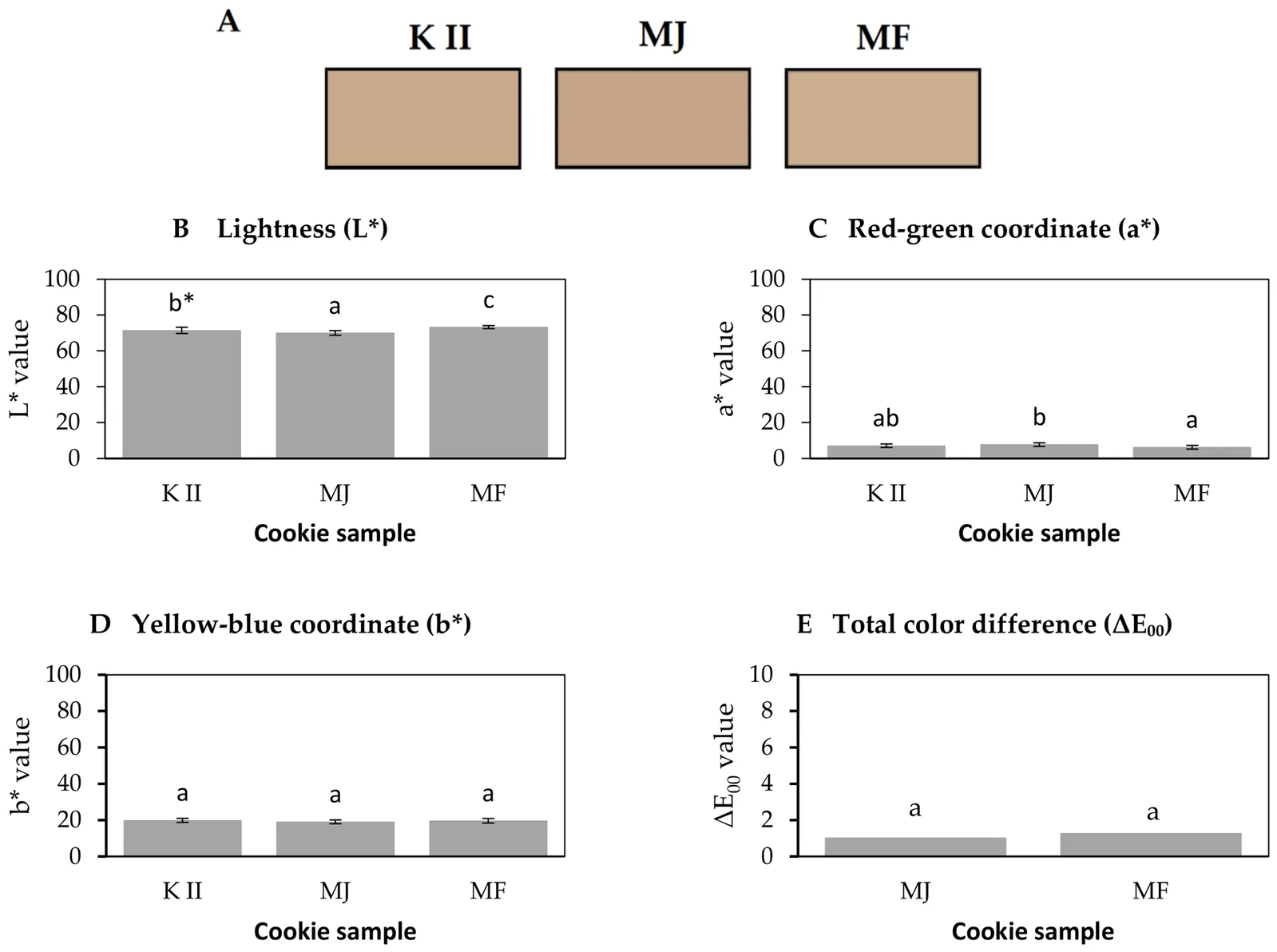
Color parameters of the cookie samples analyzed in Stage II. (**A**) Visualization of sample colors in RGB color space based on the mean CIELAB L*, a*, and b* values. (**B**–**D**) Values of the L*, a*, and b* color parameters. (**E**) Total color difference (Δ*E*_00_) calculated relative to the control sample. *—values are presented as mean ± standard deviation (*n* = 10). Different letters above the bars indicate statistically significant differences among samples according to Tukey’s test (*p* ≤ 0.05).

Color analysis showed that cookies containing maltodextrin obtained from either light-fleshed or purple-fleshed potato starch differed only slightly from the control cookies, and these differences were classified as barely perceptible (Figure 6). The MF cookies showed a slightly greater perceptual color difference (Δ*E*_00_), mainly because of their higher lightness (L*); however, this difference was not statistically significant.

**Figure 7:**
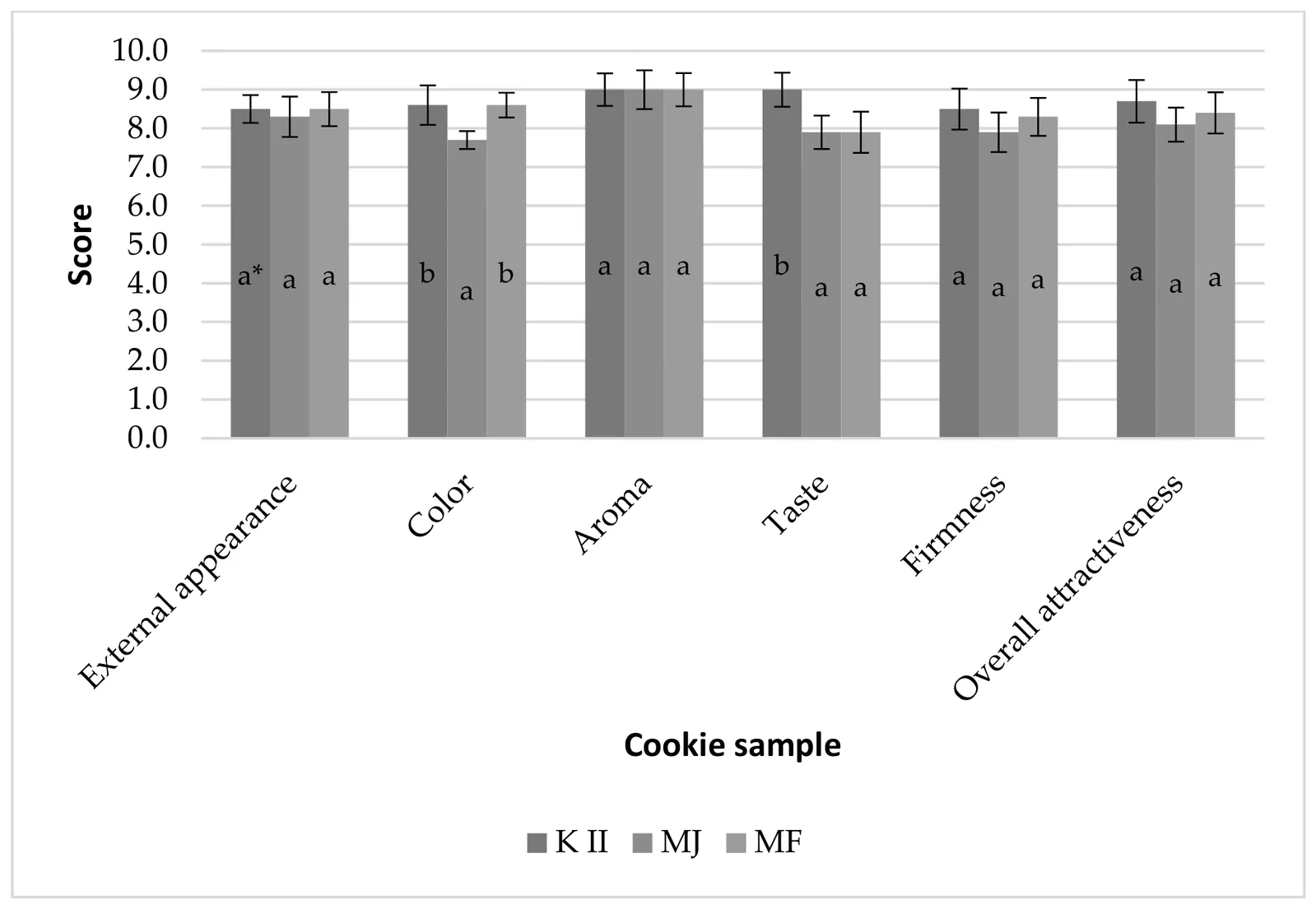
Sensory evaluation of the cookie samples analyzed in study Stage II. *—Values are presented as mean ± SD (*n* = 14). Different letters indicate statistically significant differences according to Tukey’s test (*p* < 0.05).

Consumer evaluation showed that the use of maltodextrin obtained from light-fleshed potato starch (MJ) or purple-fleshed potato starch (MF) did not substantially reduce cookie acceptability. All samples received the maximum aroma score of 9.0 points. Sample MF received the same appearance and color scores as the control sample, whereas MJ received slightly lower scores for color, flavor, and consistency. The control sample (K II) received the highest overall appeal score of 8.7 points, followed by MF at 8.4 points and MJ at 8.1 points. These high scores confirmed the good consumer acceptance of all variants (Figure 7).

Considering the texture, color, and sensory properties of the Stage II cookies, no substantial overall differences in quality were observed between the control and the samples containing maltodextrin obtained from the two types of potato starch. Both maltodextrins were therefore retained as separate variants in Stage III to determine whether differences would be observed in the bioactive compound profile, particularly the polyphenol composition, and to examine their potential interactions with FPS.

### 2.3. Effect of the FPS Incorporation Level on Polyphenol Content and Individual Phenolic Compounds (Determined by HPLC), and the Antioxidant Activities of Partially Fat-Replaced Cookies

The flaxseed press cake (*Linum usitatissimum* L.) used in study Stage III contained substantial amounts of bioactive compounds and exhibited high antioxidant potential. The Total Phenolic Content (TPC), determined after extraction with 80% ethanol using the Folin–Ciocalteu method, was 400.67 mg catechin/100 g DM (202.33 mg gallic acid –GAE/100 g DM; 2.02 mg GAE/g DM). The raw material also contained flavonoids (87.32 mg rutin/100 g DM), flavonols (75.43 mg quercetin/100 g DM) and phenolic acids (409.13 mg ferulic acid/100 g DM). The antioxidant activity of the FPS was 79.88 and 26.71 microM Trolox/kg DM in the ABTS and DPPH assays, respectively. Comparable values for total polyphenol content in FPS have been reported by other authors, and the results obtained in the present study are generally consistent with those described in the literature. Stodolak et al. [38] determined 3.58 mg GAE/g DM in a buffer extract and 8.50 mg GAE/g DM following extraction with an acetone–water mixture. Alu’datt et al. [39] obtained values of approximately 2.0–2.5 mg GAE/g DM, whereas Terpinc et al. [40] reported 0.12 mg GAE/g DM in a 96% ethanol extract and 0.90 mg GAE/g DM following extraction with 70% methanol. Teh et al. [41] further demonstrated that the efficiency of polyphenol recovery from defatted oilseed cakes, including FPS, strongly depends on the solvent system used, with aqueous mixtures of organic solvents generally providing greater extraction efficiency than pure solvents [41]. Therefore, although the results of the present study are generally consistent with previous findings, differences between the reported values should be interpreted with caution. Such discrepancies may largely arise from methodological differences, particularly the type and concentration of the extraction solvent, solvent polarity, extraction time, temperature, and solid-to-solvent ratio, all of which can markedly affect the efficiency and selectivity of phenolic compound extraction [41,42].

Zhang et al. [43] determined 32.00–78.01 mg GAE/g extract and 1.42–2.73 mg RE/g extract in FPS obtained using different oil extraction methods. The variation in these values was mainly attributed to the oil extraction method and extraction conditions. Most phenolic compounds remain in the residual material after pressing because only a small proportion is transferred to the oil fraction. The characteristic phenolic compounds of FPS include lignans and chlorogenic, ferulic, p-coumaric, gallic, and p-hydroxybenzoic acids [24,43,44]. These results indicate that FPS served not only as a partial flour replacer but also as an important source of bioactive compounds in the B1–B3 cookie formulations.

Study stage III was the main experimental stage. At the previously selected level of margarine replacement with maltodextrin (10%), FPS was added at levels of 10%, 20%, and 30% to formulations containing either of the two types of maltodextrin. The bioactive properties of the resulting cookies were evaluated by determining their polyphenol content, including the phenolic compound profile obtained by HPLC, and their antioxidant potential.

The Total Phenolic Content (TPC) of the cookies was determined using two independent spectrophotometric methods: the Folin–Ciocalteu (F–C) method described by Singleton et al. [45] and a method without the F–C reagent described by Mazza et al. [46] with modifications Oomah et. al. [47]. The use of two methods was justified by the limited selectivity of the F–C reagent, which can react not only with polyphenols but also with other reducing substances, including amino acids, proteins, saccharides, and Maillard reaction products. These compounds may be present in substantial amounts, particularly in baked products such as cookies [48,49].

The results showed that the incorporation of 10% FPS (B1J and B1F) increased the polyphenol content compared with the control sample, regardless of the analytical method used (Table 3). When determined using the F–C reagent, the polyphenol contents of B1J and B1F were more than five and six times higher, respectively, than that of the control. When the method without the F–C reagent was used, the corresponding values were approximately three and four times higher. The highest polyphenol contents were recorded in samples containing 30% FPS, namely B3J and B3F. The values determined using the F–C reagent were 416.04 and 424.11 mg catechin/100 g DM, respectively, while those obtained without the F–C reagent were 165.40 and 165.77 mg catechin/100 g DM. The results obtained using the F–C reagent represented an increase of more than 13-fold compared with the control sample.

Cookies containing maltodextrin obtained from purple-fleshed potato starch (BXF samples) also had higher polyphenol contents than the corresponding variants containing maltodextrin obtained from light-fleshed potato starch (BXJ samples). These differences were particularly pronounced at the 10% FPS incorporation level (Table 3). The observed differences could potentially be associated with phenolic compounds originating from the potato tubers used to produce the maltodextrins; however, this hypothesis cannot be confirmed because the phenolic composition of the maltodextrins was not directly determined. In plant materials, polyphenols can interact with macromolecules, particularly polysaccharides, through hydrogen bonding and hydrophobic interactions [50,51]. Covalent bonds and ionic interactions, including those between anthocyanins and pectins, have also been reported [52,53]. The ability of polyphenols to bind to polysaccharides may also increase with their molecular weight [50,54,55]. Phenolic compounds may also interact with starch, which is converted into maltodextrin through enzymatic hydrolysis. The polyphenol content of colored-fleshed potatoes ranges from 53 to 177 mg/100 g and is generally higher than that of light-fleshed potatoes [56]. A similar relationship has been reported for phenolic acids. Potato tubers contain chlorogenic acid and its derivatives, including neochlorogenic and cryptochlorogenic acids, as well as gallic, ferulic, protocatechuic, caffeic, cinnamic, sinapic, and p-coumaric acids. Their contents in red- or purple-fleshed potatoes may be two to four times higher than those found in light-fleshed cultivars [57,58]. Lewis et al. [59] also found that red- and purple-fleshed potatoes contained approximately twice as many flavonoids as light-fleshed cultivars. Anthocyanins are particularly important because they are a subclass of flavonoids responsible for the red or purple color of potato flesh and skin. Their content in colored potato cultivars may range from 5.5 to 35 mg/100 g, whereas these compounds are generally absent from light-fleshed potatoes [60]. Although these findings provide a possible explanation for the differences observed between the BXF and BXJ variants, the phenolic composition of the maltodextrins used in the present study was not directly analyzed. Therefore, any contribution of maltodextrin-derived phenolic compounds to the phenolic profile of the cookies remains hypothetical and cannot be confirmed based on the present results.

Man et al. [61] found that the incorporation of flaxseed flour to biscuits significantly increased their polyphenol content. A 40% incorporation increased the polyphenol content by 25% compared with the control sample, whereas the lowest incorporation level of 10% resulted in an 11% increase. Plustea et al. [62] also reported an increase in polyphenolic compounds in cookies enriched with lupin sprouts, which resulted in an almost tenfold increase compared with the control. A similar relationship was reported by Ertaş and Asian [63], who investigated the effects of raw and roasted hemp flour on cookie quality. The incorporation of 20% roasted hemp flour resulted in an up to fourfold increase in polyphenol content.

Flavonoid content, expressed as mg rutin/100 g DM, increased significantly in all samples enriched with FPS compared with the control. An approximately 3.5-fold increase was observed in both B3J and B3F. Samples containing maltodextrin obtained from purple-fleshed potato starch had slightly higher flavonoid contents than the corresponding samples containing maltodextrin obtained from light-fleshed potato starch, although these differences were not statistically significant (Table 3). Gumul et al. [64] reported a similar increase in the flavonoid content of muffins enriched with FPS. The incorporation of 20% and 30% FPS increased flavonoid content by approximately 120% and 130%, respectively, compared with the control.

Flavonols were not detected in the control sample, whereas enrichment with FPS resulted in a gradual increase in their content, particularly in samples containing maltodextrin obtained from purple-fleshed potato starch. The highest flavonol content was determined in B3F and B3J, with both samples containing 2.77 mg quercetin/100 g DM.

For phenolic acids determined spectrophotometrically, enrichment with FPS in cookies resulted in a clear increase in their content compared with the control sample, in which these compounds were not detected. As the proportion of FPS increased from 10% to 30%, the phenolic acid content increased approximately 3.1-fold in the J variants and 2.8-fold in the BXF variants. At each incorporation level, higher values were obtained for samples containing maltodextrin obtained from purple-fleshed potato starch. Compared with the corresponding BXJ variants, the phenolic acid contents of B1F, B2F, and B3F were approximately 25%, 37%, and 12% higher, respectively. These results suggest that the increase in phenolic acid content in the final product was primarily associated with the increasing proportion of FPS. The type of maltodextrin may have additionally affected the retention or extractability of these compounds, particularly at FPS incorporation levels of 10% and 20%.

The spectrophotometric determinations were complemented by HPLC analysis of phenolic compounds in FPS and the cookies containing it. The raw FPS contained ferulic acid (0.87 mg/100 g DM), 4-hydroxybenzoic acid (1.11 mg/100 g DM), sinapic acid (2.18 mg/100 g DM), and gallic acid (1.67 mg/100 g DM), among other compounds. These values were within the ranges reported in the literature for FPS and flaxseed meal. The gallic acid content of the analyzed raw material (1.67 mg/100 g DM) was similar to the values reported by Kaur et al. [65] and Carrà et al. [66], while its ferulic acid content (0.87 mg/100 g DM) corresponded to the value reported by Kaur et al. [65]. Krimer Malešević et al. [67] also identified gallic and 4-hydroxybenzoic acids in FPS, although their concentrations depended on the fraction analyzed. Differences among published results may be attributed primarily to the extraction procedure and whether free, bound, and esterified forms of phenolic acids were included in the analysis [41,42]. 

Comparison of the cookie variants containing maltodextrin obtained from purple-fleshed potato starch (BXF) and light-fleshed potato starch (BXJ) revealed differences in the phenolic compound profiles determined by HPLC. In all variants, enrichment with FPS increased the total content of the identified phenolic compounds compared with the control sample. In the BXF variants, the total content increased approximately 4.8-, 6.2-, and 6.6-fold in B1F, B2F, and B3F, respectively, compared with K III. The corresponding BXJ variants also showed an increase in the total content of identified phenolic compounds with increasing FPS incorporation. At the 10% and 20% FPS incorporation levels, the total contents of the identified compounds in the BXF variants were approximately 119% and 123% higher, respectively, than those in the corresponding BXJ samples, whereas at the 30% incorporation level, the total content in B3F was approximately 12% higher than that in B3J (Table 4 and Table 5). The progressively smaller difference between the BXF and BXJ variants with increasing FPS incorporation suggests that the contribution of phenolic compounds derived from FPS became increasingly dominant, thereby reducing the relative influence of the type of maltodextrin on the overall phenolic compound profile. This trend was confirmed both by the direct comparison of cookie variants containing maltodextrin obtained from purple-fleshed potato starch (BXF) and light-fleshed potato starch (BXJ) (Table 6), and by the heatmap analysis based on log_2_ fold-change values relative to the control sample (Table 7). The total content of the identified phenolic compounds was higher in the BXF variants than in the corresponding BXJ variants at all levels of FPS incorporation, with statistically significant differences observed for B1F vs. B1J and B2F vs. B2J (*p* < 0.001) and for B3F vs. B3J (*p* < 0.05) (Table 6). The log_2_ FC values calculated for the total content of the identified compounds were approximately 2.274, 2.623, and 2.721 for B1F, B2F, and B3F, respectively, and approximately 1.005, 1.387, and 2.499 for B1J, B2J, and B3J, respectively. Thus, the difference between the BXF and BXJ variants was particularly pronounced at the lower levels of FPS incorporation, whereas it decreased as the proportion of press cake increased. This may indicate that, at lower FPC levels, the characteristics of the maltodextrin used played a greater role in shaping the final phenolic profile of the cookies, whereas at the 30% incorporation level, phenolic compounds originating from FPS contributed increasingly to the overall pool of phenolic compounds. The influence of maltodextrin type was also clearly evident for individual phenolic acids. At the 20% and 30% levels of FPS incorporation, the BXF variants contained significantly higher amounts of 3,4-dihydroxybenzoic acid, neochlorogenic acid, and chlorogenic acid than the corresponding BXJ variants (*p* < 0.001). The difference was particularly pronounced for chlorogenic acid: all BXF variants showed an increase relative to the control (log_2_ FC = 2.090, 2.133, and 2.010 for B1F, B2F, and B3F, respectively), whereas all BXJ variants showed lower values than the control (log_2_ FC = −0.478, −0.186, and −0.630). The largest positive relative changes in B3F were observed for neochlorogenic acid and 3,4-dihydroxybenzoic acid, with log_2_ FC values of 4.629 and 4.292, respectively. It should also be noted that at the lowest level of FPS incorporation, neochlorogenic acid content was significantly higher in B1J than in B1F (*p* ≤ 0.01), indicating that the effect of maltodextrin type on the profile of individual phenolic compounds also depended on the level of product enrichment.

The higher values observed in the F variants were particularly evident for 3,4-dihydroxybenzoic (protocatechuic), chlorogenic, and neochlorogenic acids. Chlorogenic acid and its isomers are among the predominant phenolic compounds in potatoes, particularly in purple-fleshed cultivars from which the starch used to produce the maltodextrin was obtained [58,68,69]. At the highest FPS incorporation level, the chlorogenic acid content of B3F was approximately 2.1-fold higher than that of B3J, while the neochlorogenic acid content was approximately 1.9-fold higher. An even greater difference was observed for protocatechuic acid. This compound may also have originated from the FPS, which could have contributed to its increasing content, particularly in samples B1F–B3F [15,70].

At the 10% FPS incorporation level, the gallic acid content of B1F was approximately 2.6-fold higher than that of B1J, while at the 20% incorporation level, it was approximately 1.9-fold higher in B2F than in B2J. At the 30% incorporation level, this relationship was reversed, and B3J contained approximately 11% more gallic acid than B3F. This may suggest that the type of maltodextrin had a greater influence at lower FPS incorporation levels, whereas the flaxseed component became the predominant factor at the highest incorporation level. Gallic acid is naturally present in potato tubers, with generally higher phenolic acid contents reported for colored-fleshed than for light-fleshed cultivars [71]. Therefore, the potato raw material used for starch isolation may have contributed to the differences observed between the F and J variants. However, the high gallic acid content of the final cookies cannot be explained solely by its direct contribution from the FPS. A possible explanation may involve the thermally induced release of gallic acid from bound and galloylated forms and an increase in its extractability during baking; however, these mechanisms were not directly investigated in the present study. Kim et al. [72] demonstrated the formation of free gallic acid during the thermal hydrolysis of tannic acid, while Kaur et al. [73] observed changes in the content of bound gallic acid in FPS flour following intensive thermal treatment.

Changes were also observed in the ferulic acid content. This compound may originate from both FPS and wheat flour, in which it occurs mainly in forms bound to cell-wall arabinoxylans [74]. The increase in its measured content may therefore have resulted from both its direct contribution from the raw materials and the enhanced release of bound forms during baking, as previously observed in cereal products [75]. Ferulic acid can also readily undergo decarboxylation during baking [76].

Sinapic acid showed a different pattern. Following enrichment, its content in the BXF variants was approximately 83% lower than that in the control, despite the relatively high content of this compound in the raw FPS. This may indicate lower thermal stability, chemical transformation, or reduced extractability in the final product. During heating, sinapic acid may undergo decarboxylation to 4-vinylsyringol (canolol), among other reactions [76]. Kaur et al. [73] also confirmed that intensive thermal treatment may either increase or decrease the contents of individual phenolic compounds in FPS.

The varying changes in individual phenolic acids indicate that the final HPLC profile of the cookies resulted not only from their direct contribution from the raw materials but also from their release from bound forms, thermal transformations, retention, and changes in extractability during baking.

A two-way ANOVA was additionally performed for the FPS-containing variants to evaluate the effects of FPS incorporation level, maltodextrin type, and their interaction on the phenolic acid profile. Both FPS incorporation level and maltodextrin type significantly affected the total content of identified phenolic acids (*p* < 0.001), and a significant FPS level × maltodextrin type interaction was also observed (*p* < 0.001). Significant effects of both factors were found for all eight individual phenolic acids analyzed. The interaction between FPS level and maltodextrin type was significant for seven of the eight phenolic acids (*p* < 0.05), with chlorogenic acid being the only exception (*p* = 0.327). These results indicate that the phenolic acid profile was influenced by both FPS incorporation level and maltodextrin type, with significant interactions between these factors for most compounds. Detailed results of the two-way ANOVA are presented in Table S2.

**Figure 8:**
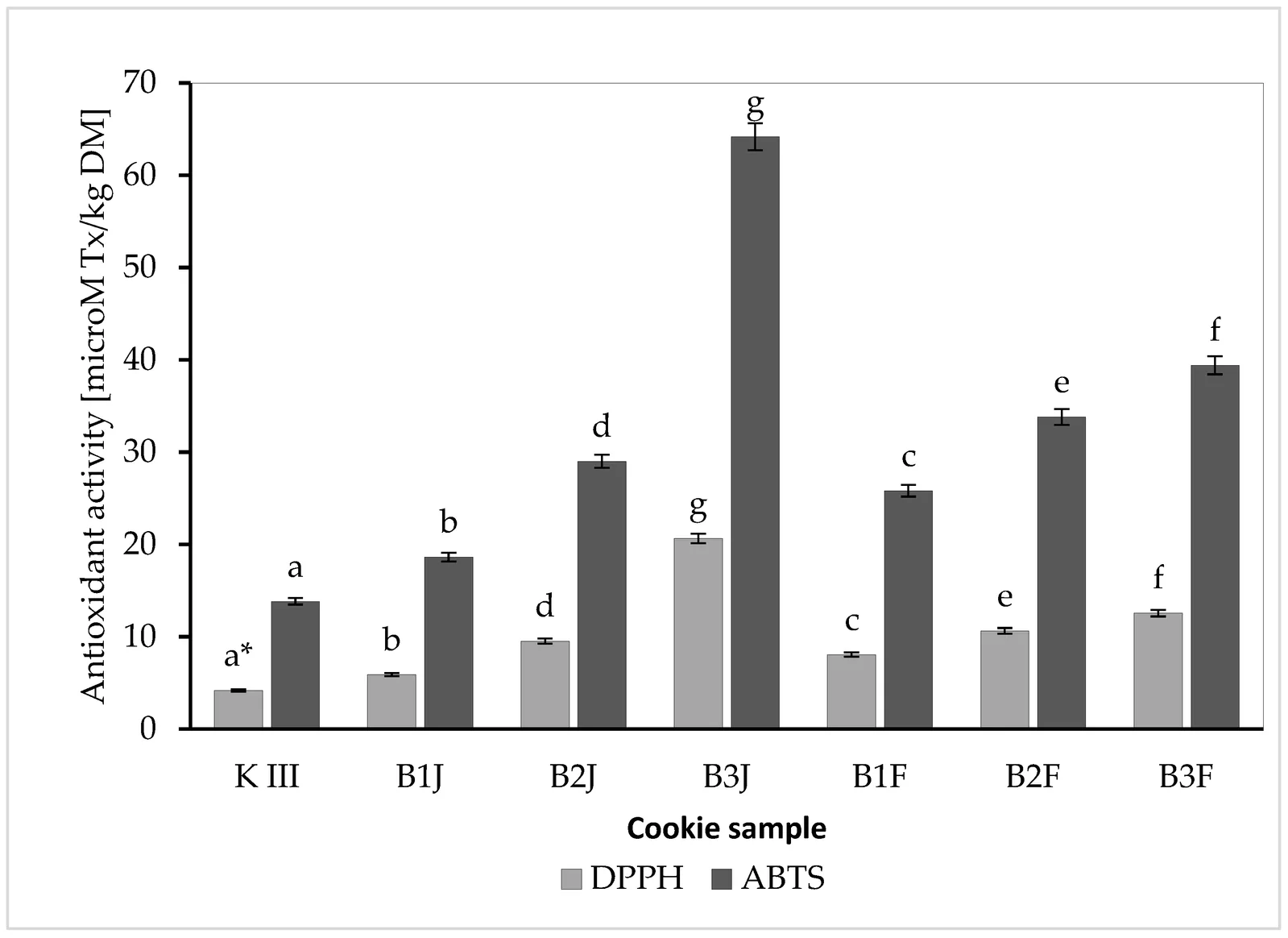
Antioxidant activity determined using the DPPH and ABTS assays in the cookie samples analyzed in Stage III. Values are presented as mean ± standard deviation (*n* = 4). *—different letters above the bars indicate statistically significant differences according to Tukey’s test (*p* < 0.05).

Antioxidant activity was subsequently determined using two widely applied assays, ABTS and DPPH. This activity is largely associated with the phenolic compounds described above (Table 7) [77,78]. The results were expressed as Trolox equivalents (Tx) in microM Tx/kg DM. Both assays measure antioxidant activity, but they differ in their reaction mechanisms and sensitivity to different classes of antioxidants. Their combined use therefore provides a more comprehensive assessment of the antioxidant properties of the analyzed products. The results obtained for the Stage III samples showed that the polyphenolic compounds, contributed mainly by the FPS, were associated with the high radical-scavenging and antioxidant activity of the enriched cookies. The control sample (K III) exhibited the lowest antioxidant activity, with values of 13.84 microM Tx/kg DM in the ABTS assay and 4.19 microM Tx/kg DM in the DPPH assay. Antioxidant activity increased proportionally with the FPS incorporation level (Figure 8). The highest activity was recorded for B3J, reaching 64.17 microM Tx/kg DM in the ABTS assay and 20.65 microM Tx/kg DM in the DPPH assay. These values were approximately 4.6- and 4.9-fold higher, respectively, than those of the control sample. The increase in antioxidant activity in B3J was strongly correlated with the previously determined contents of phenolic compounds. Total polyphenol content was correlated with both DPPH and ABTS activity (r = 0.87). Flavonoid content was correlated with DPPH and ABTS activity at r = 0.87 and r = 0.86, respectively, while phenolic acid content was correlated with both assays at r = 0.85. High antioxidant activity was also recorded for B3F and B2F. Their ABTS values were 39.4 and 33.8 microM Tx/kg DM, respectively, while their DPPH values were 12.6 and 10.6 microM Tx/kg DM, respectively.

The values obtained using the ABTS assay were consistently higher than those obtained using the DPPH assay. This difference may be attributed to the broader applicability of the ABTS assay, which can measure the activity of both hydrophilic and lipophilic antioxidants. In contrast, the DPPH assay is more selective and is primarily suitable for compounds that are readily soluble in organic solvents [79,80].

Man et al. [61] showed that the incorporation of flaxseed flour (RFSF) to biscuits significantly increased their antioxidant activity measured using the DPPH radical. This increase was proportional to the amount of flaxseed flour used in the formulation. The highest antioxidant activity was recorded for B40, which reached a radical-scavenging activity (RSA) of 32.03%, whereas the control sample had the lowest value of 13.67% RSA. This effect was attributed to the presence of potent antioxidants naturally occurring in flaxseed, particularly lignans such as secoisolariciresinol diglucoside (SDG), which is also present in flaxseed oil. Significant increases in the antioxidant properties of cookies and muffins containing flaxseed flour or FPS were also reported by Kaur et al. [81] and Gumul et al. [64].

### 2.4. Effect of FPS Incorporation Level on the Quality and Physical Properties of Partially Fat-Replaced Cookies

**Figure 9:**
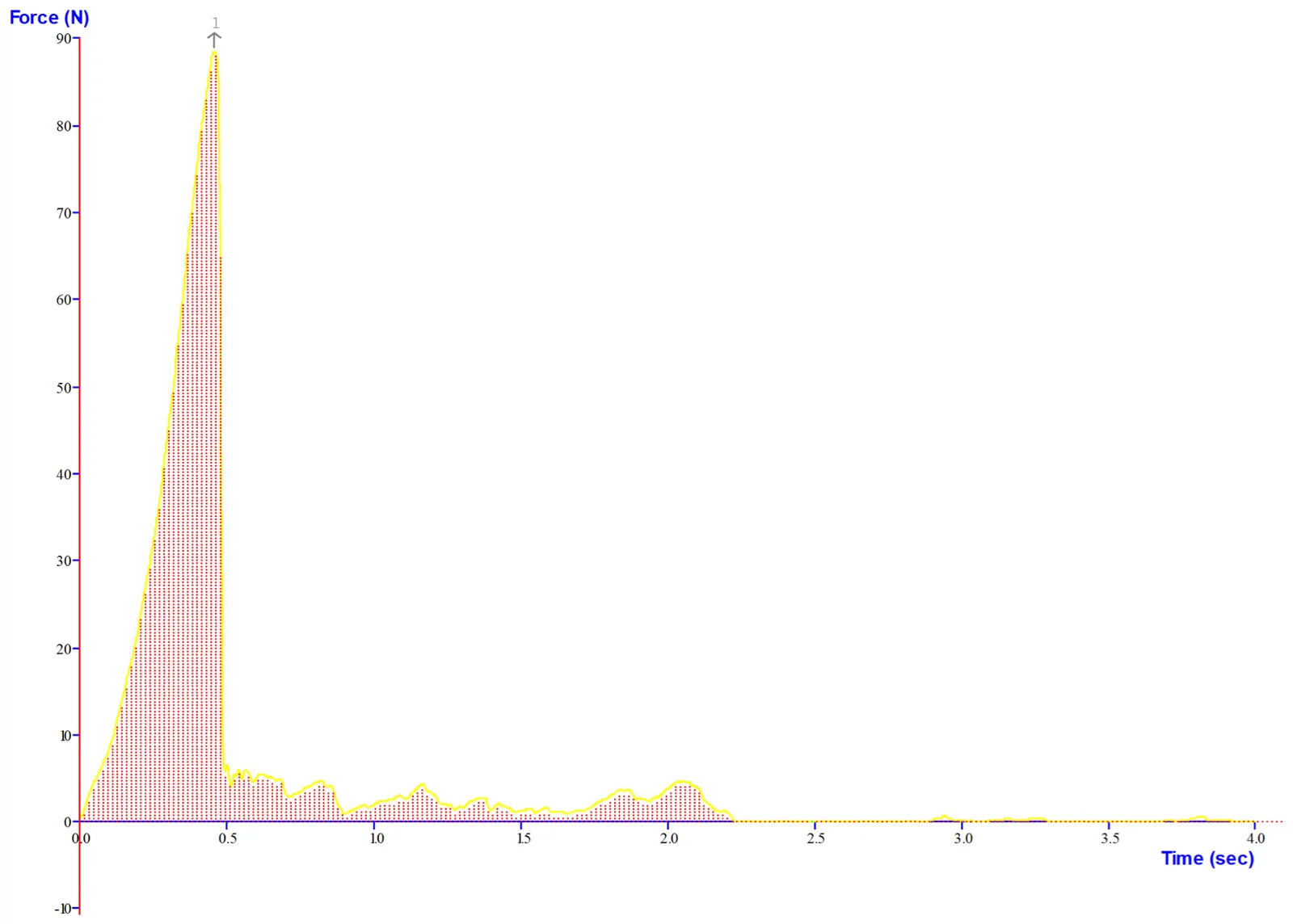
Representative force (N) versus time (s) curve obtained during texture analysis of the B3J cookie using a texture analyzer.

Table 8 presents the hardness of the cookies, expressed in newtons [N], and their cutting energy [N·s] on the day of baking and after 30 days of storage under controlled conditions. A representative force–time curve recorded during the texture analysis of sample B3J is shown in Figure 9. The maximum force corresponds to cookie hardness, while the area under the curve represents the cutting energy (Table 9).

On the Day of Baking

The control sample (K III) had the lowest hardness (25.24 ± 3.20 N). The incorporation of FPS increased cookie hardness, which reached more than three times the control value in B2J (78.39 ± 1.80 N). An overall tendency toward increasing hardness with increasing FPS incorporation was also observed. The type of potato starch used to obtain the maltodextrin affected the mechanical properties of the cookies, particularly in the variants containing maltodextrin obtained from purple-fleshed potato starch. However, this effect was less pronounced than that of the FPS itself. The increase in hardness may be attributed to the greater water absorption capacity of the formulation containing FPS and competition between this ingredient and gluten for available water. Ajila et al. [82] proposed a similar explanation for products enriched with mango peel flour. Srivastava et al. [83] also reported increases in cookie hardness ranging from 3% to 17% compared with the control when pomegranate peel flour was used as an enriching ingredient.

For cutting energy, no statistically significant differences were observed between most of the enriched samples and the control. The high standard deviations resulted in considerable overlap among the confidence intervals. Cutting energy in the samples containing FPS ranged from 23.39 ± 7.81 N·s in B2F to 31.90 ± 8.41 N·s in B2J. These results indicate that FPS did not significantly increase the resistance of the cookies to cutting. However, the high variability of the results indicates that further research is required. Kidoń et al. [84] reported similar findings, with no differences in the force required to fracture wafers enriched with 10% or 12.5% apple press cake.

After 30 Days of Storage:

After 30 days of storage under controlled conditions, only minor changes in cookie texture were observed. The control sample (K III) showed a statistically nonsignificant decrease in both hardness and cutting energy. Hardness increased by approximately 20% in B1J and 39% in B1F compared with the values measured on the day of baking. These increases may have resulted from gradual moisture loss or changes in the starch structure. Some stiffening of the cookie structure was also reflected in the higher cutting energy, particularly in B1J. Hardness in B3J, B2F, and B3F increased by approximately 10–20%, while their cutting energy remained similar to the values recorded on the day of baking. Overall, most samples showed only minor textural changes after 30 days, while the absence of significant changes in cutting energy in many samples indicated good mechanical stability during storage (Table 8).

**Figure 10:**
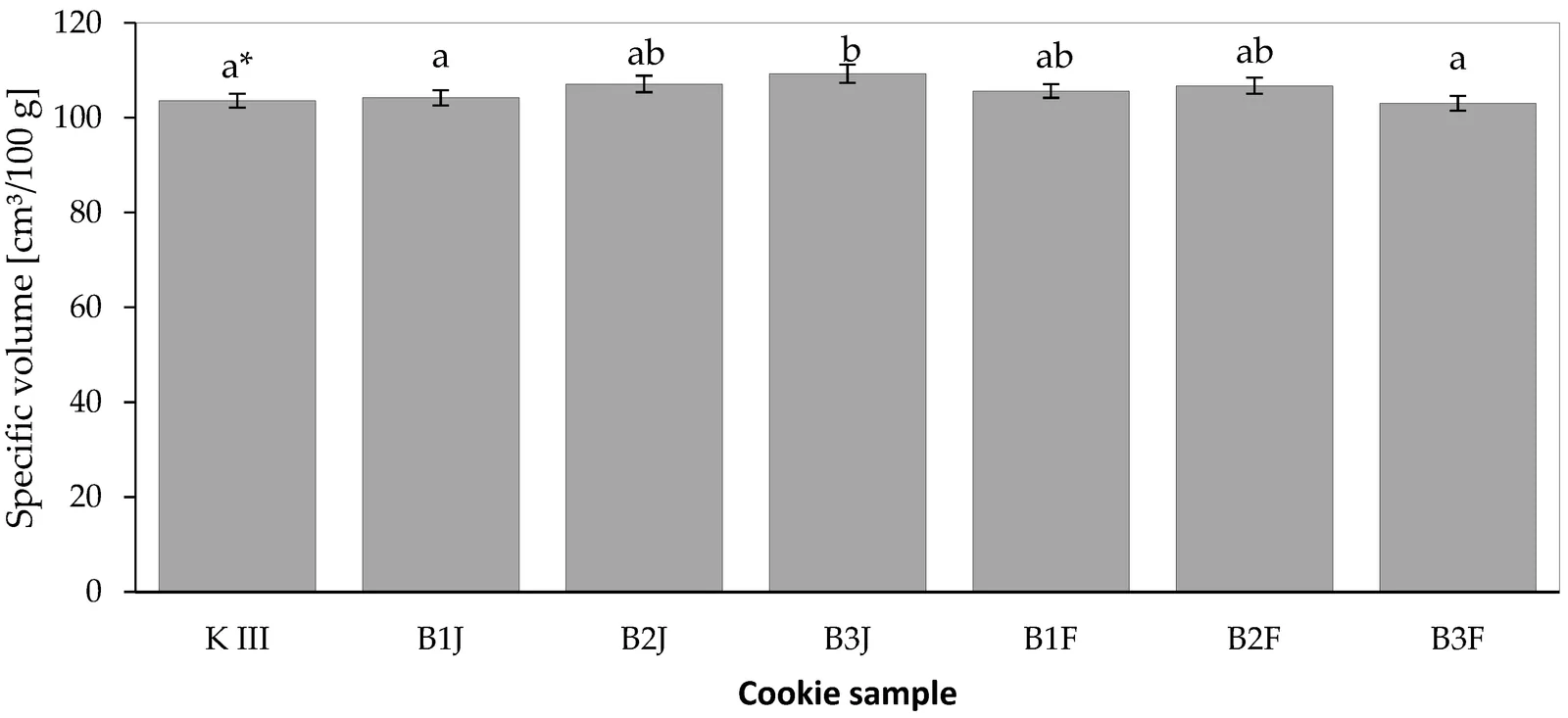
Specific volume of the cookie samples analyzed in Stage III. *—Values are presented as mean ± standard deviation (*n* = 5), different letters above the bars indicate statistically significant differences according to Tukey’s test (*p* < 0.05).

The specific volumes of the cookies containing FPS were generally similar to that of the control sample (Figure 10). However, among the cookies containing maltodextrin obtained from light-fleshed potato starch, specific volume tended to increase with increasing FPS incorporation. The greatest increase relative to the control, amounting to 5.5%, was recorded for B3J (109.28 ± 1.31 cm^3^/100 g), which contained maltodextrin obtained from light-fleshed potato starch and 30% FPS. This finding is noteworthy because FPS is rich in dietary fiber, and partial replacement of wheat flour with fiber-rich material may disrupt the continuity of the starch–protein matrix and interfere with gas-cell development and stability. Insoluble fiber particles may restrict gas-cell expansion and promote their destabilization, particularly at higher incorporation levels. This result may indicate that maltodextrin partially compensated for these structural effects of FPS incorporation. In addition to acting as a fat replacer, maltodextrin can also function as a bulking and structure-forming agent, modifying water distribution, viscosity, and the rheological properties of the continuous phase. Maltodextrin obtained from light-fleshed potato starch may also have promoted the formation of a denser matrix with greater resistance to cutting [85]. Its contribution to matrix cohesiveness may have favored the stabilization and retention of air incorporated during mixing and gases generated during baking, thereby helping to preserve gas cells until structural setting. It is possible that maltodextrin affected the distribution of flaxseed fibers and pore formation; however, these structural mechanisms were not directly investigated [86,87]. Cookies containing maltodextrin obtained from purple-fleshed potato starch showed a different pattern. At the highest FPS incorporation level of 30%, B3F had a specific volume of 103.05 ± 0.35 cm^3^/100 g, which was slightly lower than that of the control sample. This suggests that the extent to which maltodextrin compensated for FPS-induced structural changes may have depended on its physicochemical properties and source. At higher incorporation levels, FPS combined with maltodextrin obtained from purple-fleshed potato starch adversely affects dough structure, possibly by obstructing pore formation or increasing viscosity and thereby limiting cookie expansion during baking [88,89]. Because dough rheology and microstructure were not directly characterized, the proposed mechanisms should be regarded as tentative.

**Figure 11:**
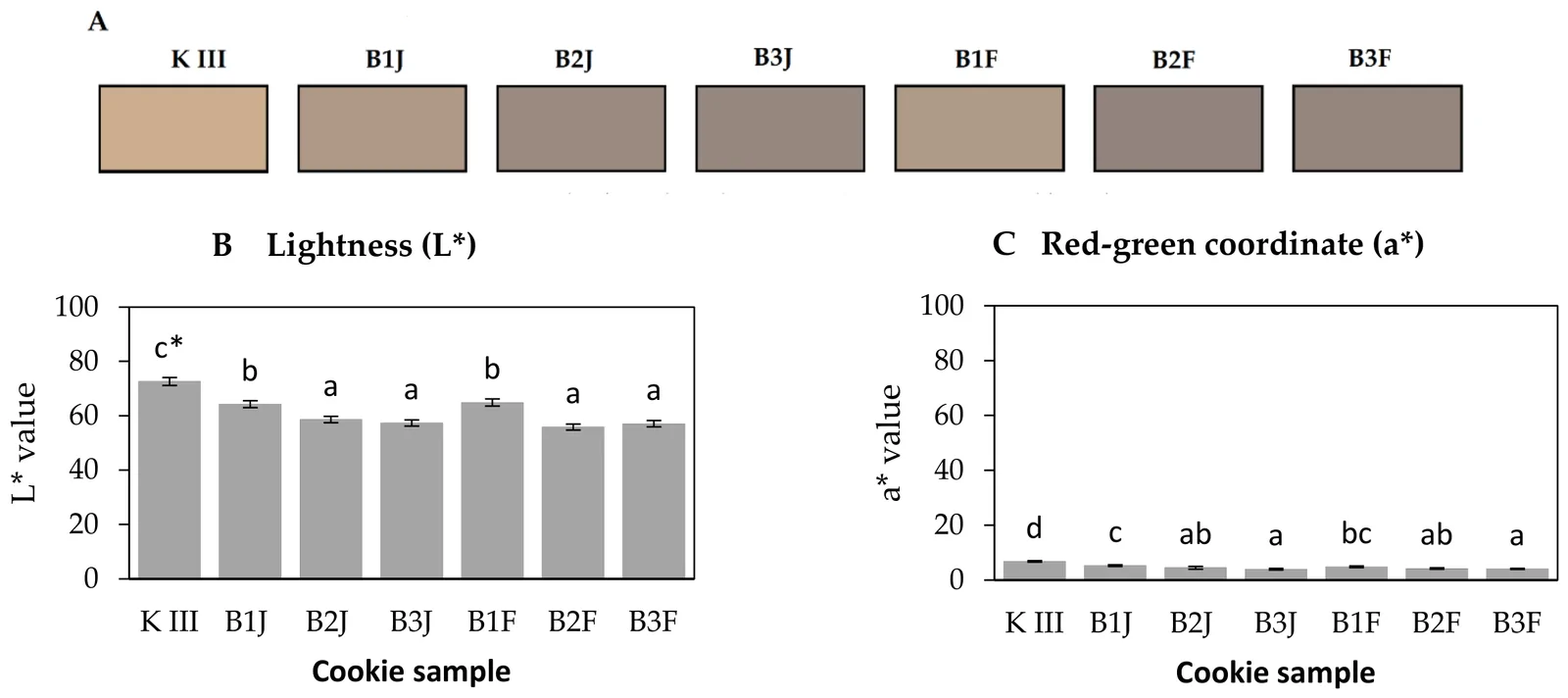

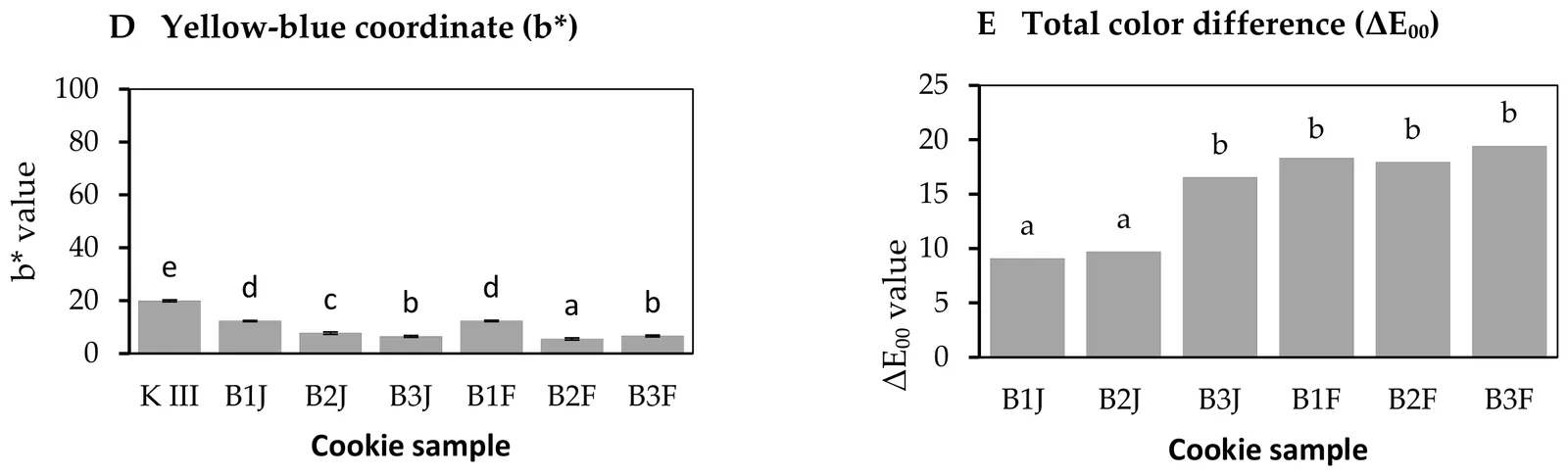
Color parameters of the cookie samples analyzed in Stage III. (**A**) Visualization of sample colors in RGB color space based on the mean CIELAB L*, a*, and b* values. (**B**–**D**) Values of the L*, a*, and b* color parameters. (**E**) Total color difference (Δ*E*_00_) calculated relative to the control sample. *—Values are presented as mean ± standard deviation (*n* = 10). Different letters above the bars indicate statistically significant differences among samples according to Tukey’s test (*p* ≤ 0.05).

**Figure 12:**
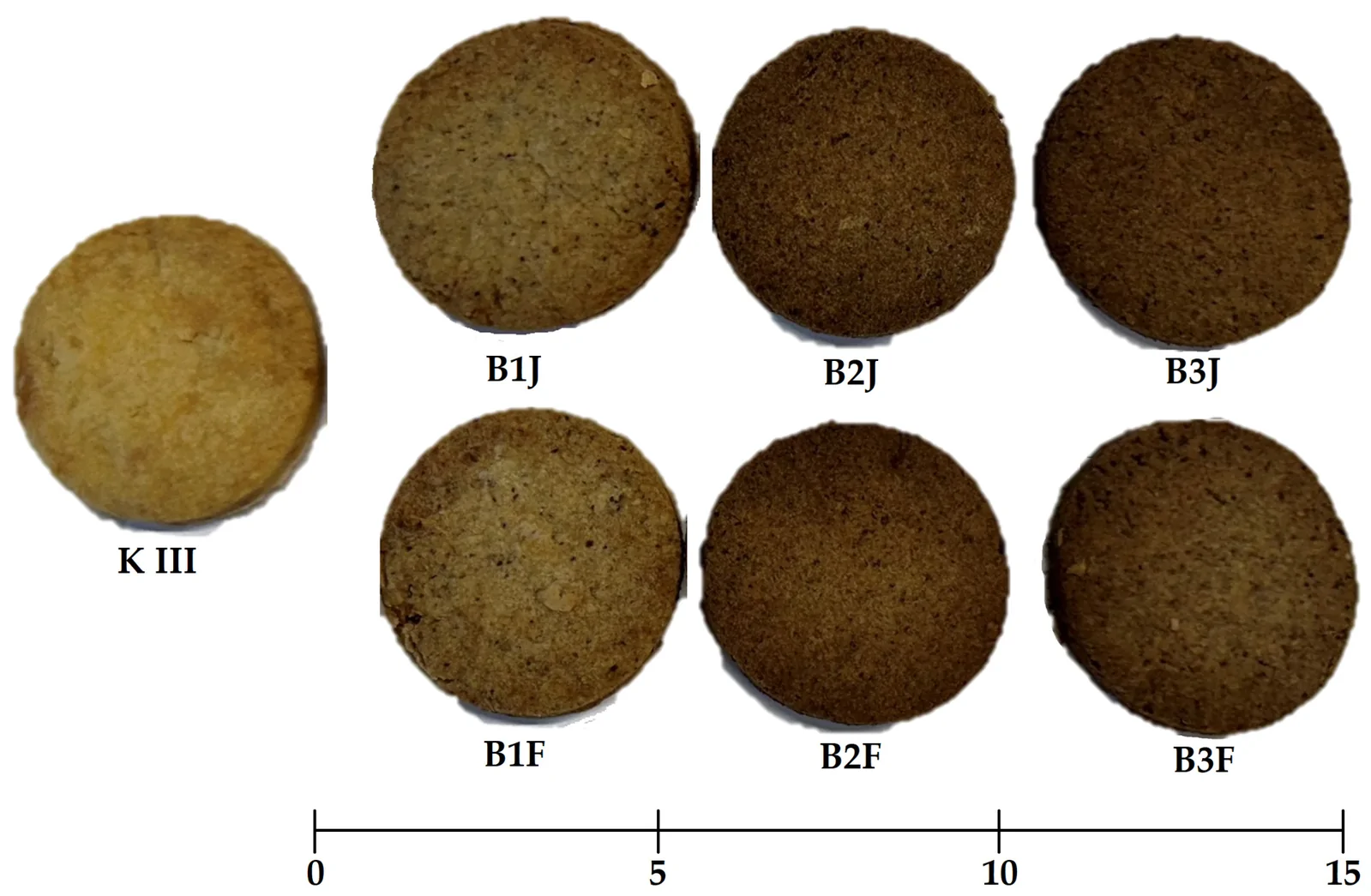
Photograph of the cookie samples analyzed in Study Stage III (photograph taken by the authors).

The color of the Stage III cookies was subsequently analyzed (Figure 11 and Figure 12). Cookies containing FPS were generally darker than the control sample (Figure 11). The control sample showed the highest lightness (L* = 72.64 ± 0.62). In samples containing maltodextrin derived from light-fleshed potato starch (B1J–B3J), the L* value progressively decreased with increasing FPS incorporation. A similar overall decrease was observed in samples containing maltodextrin derived from purple-fleshed potato starch, with B2F showing the lowest lightness (L* = 55.86 ± 0.44). Thus, the progressive darkening of the cookies was primarily associated with the increasing incorporation of FPS. Differences between the F and J variants may additionally reflect differences in the physicochemical properties of the two maltodextrins and their interactions with the other cookie ingredients. All samples had positive a* values, indicating a red color component. The control sample had the highest a* value (6.83 ± 0.33), which gradually decreased following FPS incorporation. The lowest values were recorded in the samples with the highest FPS content: B3J (3.99 ± 0.24) and B3F (4.09 ± 0.18). All samples also showed positive b* values, indicating a yellow color component. The control sample had the highest b* value (19.96 ± 0.37), whereas FPS incorporation markedly reduced this parameter. The lowest values were recorded for B2F (5.50 ± 0.51) and B3J (6.52 ± 0.69). Thus, the reduction in yellowness was mainly associated with FPS incorporation, although differences between the corresponding F and J variants indicate that the source of the maltodextrin may also have influenced the final color of the cookies. Changes in the L*, a*, and b* parameters resulted in substantial and readily perceptible color differences between the enriched cookies and the control sample (Δ*E*_00_ > 5). The largest differences relative to the control were recorded for B1F, B2F, and B3F. These results are consistent with previous studies reporting product darkening, decreases in a* and b* values, and clearly perceptible color differences between samples enriched with plant-derived materials and control samples (Δ*E*_00_ > 5) [63,90,91].

**Figure 13:**
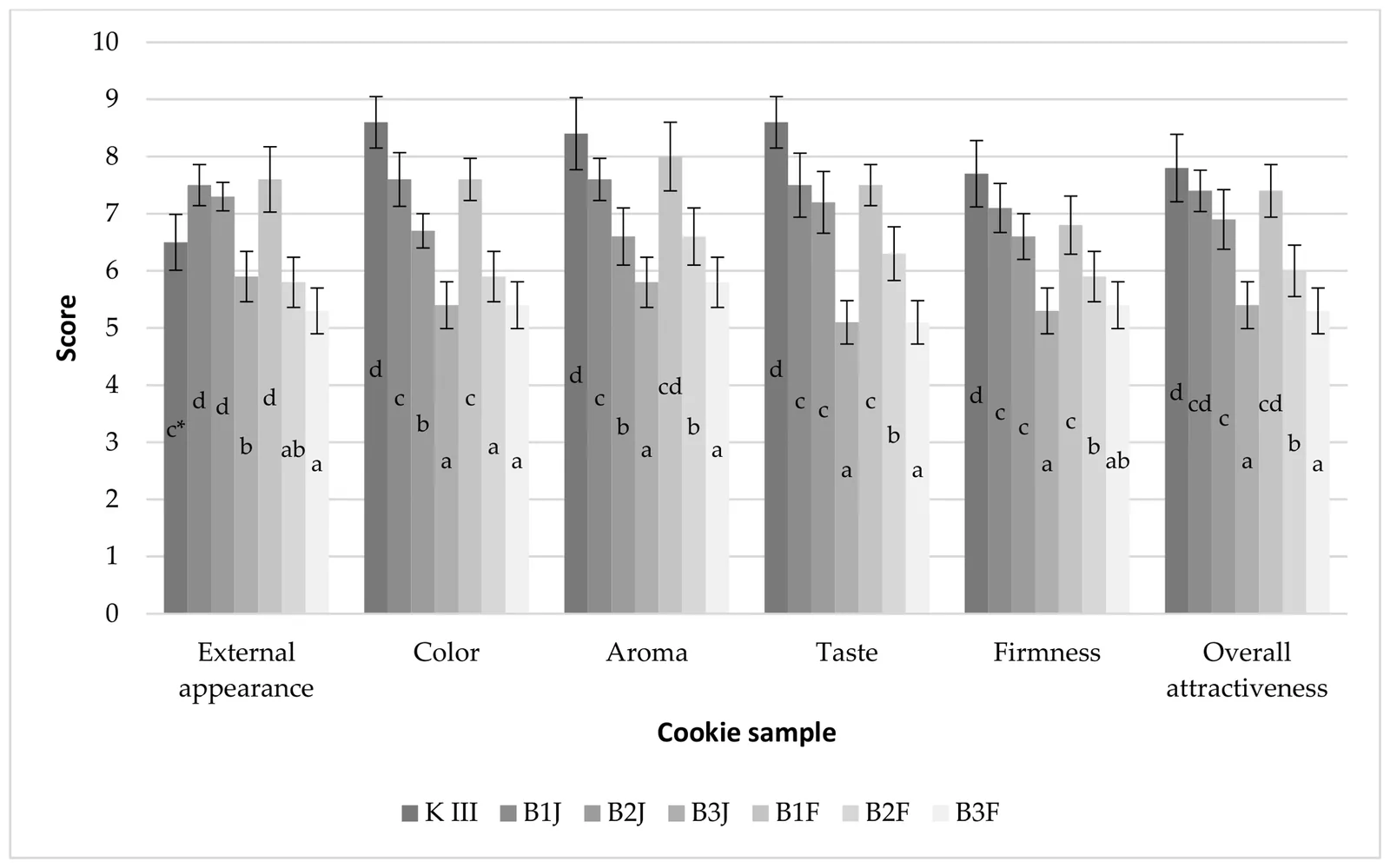
Consumer acceptance evaluation of the cookie samples analyzed in Stage III. *—Values are presented as mean ± SD (*n* = 14). Different letters indicate statistically significant differences according to Tukey’s test (*p* < 0.05).

The consumer evaluation of the cookies included six attributes: appearance, color, aroma, flavor, hardness, and overall product appeal. The control sample (K III) contained neither FPS nor maltodextrin (Figure 13). In the remaining variants, 10% of the margarine was replaced with maltodextrin obtained from either light-fleshed (BXJ) or purple-fleshed (BXF) potato starch, and FPS was added at levels ranging from 10% to 30%.

The control sample received the highest overall product appeal score of 7.8 points. Samples containing 10% FPS, B1J and B1F, also received high and identical scores of 7.4 points. Consumer acceptability gradually decreased as the proportion of FPS increased. Samples containing 20% FPS received scores of 6.9 points for B2J and 6.0 points for B2F, whereas the variants containing 30% FPS received scores of 5.4 points for B3J and 5.3 points for B3F. The lowest ratings for B3J and B3F were recorded for flavor (5.1 points for both samples), hardness (5.3 and 5.4 points, respectively), and color (5.4 points for both samples). The type of maltodextrin had less influence on the consumer evaluation of the cookies than the FPS incorporation level.

The source of the maltodextrin, obtained from either light-fleshed (J) or purple-fleshed (F) potato starch, had no significant effect on the consumer acceptability of the cookies. In both groups, the scores decreased as the proportion of FPS increased. Variants containing maltodextrin obtained from light-fleshed potato starch generally received slightly higher scores, particularly at the 20% FPS incorporation level (B2J, 6.9 points; B2F, 6.0 points). These differences may have been partly associated with the darker color of the products containing maltodextrin obtained from purple-fleshed potato starch. However, this relationship was not consistent because B1J and B1F received the same overall product appeal score of 7.4 points, while the scores for B3J and B3F were very similar at 5.4 and 5.3 points, respectively.

The control sample received the highest overall product appeal score of 7.8 points and high ratings for color, aroma, flavor, and hardness. However, B1J and B1F received higher appearance scores than the control. The variants containing 10% FPS were the most similar to the control sample. Increasing the proportion of FPS to 20%, and particularly to 30%, reduced the sensory scores, mainly for flavor, hardness, and color. This may have resulted from the characteristic intense aroma and slightly bitter aftertaste of FPS, which became more perceptible at higher incorporation levels. These results indicate that the FPS incorporation level had a greater effect on the consumer acceptability of the cookies than the type of maltodextrin used.

An important limitation of the Stage III experimental design should be acknowledged. The control sample (K III) contained neither maltodextrin nor FPS, whereas all B1–B3 variants incorporation of both maltodextrin and FPS. Consequently, differences between K III and the experimental samples cannot be attributed exclusively to FPS, as the incorporation and source of maltodextrin may also have contributed to the observed effects. Nevertheless, within each maltodextrin series (J and F), the amount of maltodextrin was kept constant while the FPS incorporation level increased from 10% to 30%. Therefore, the dose-dependent trends observed within the B1–B3 series are consistent with the effects of increasing FPS incorporation, although possible interactions between FPS and maltodextrin cannot be excluded.

## 3. Materials and Methods

### 3.1. Materials

The materials used in the study included maltodextrins with a dextrose equivalent of 17. Commercial maltodextrin (Agnex, Białystok, Poland) was used in Stage I, whereas maltodextrins prepared under laboratory conditions from potato starch were used in Stages II and III. The maltodextrins used in the experiments were produced under laboratory conditions [92] from starch isolated separately from the individual potato cultivars. The resulting maltodextrins obtained from the purple-fleshed cultivars Violetta and Blue Star were combined in a 1:1 ratio, while those obtained from the light-fleshed cultivars Lord and Tajfun were combined in a 1:1 ratio. The physicochemical, structural, and functional properties of maltodextrins derived from light-fleshed and colored potato starches were characterized in our previous study [92]. The maltodextrins showed high solubility and differed, depending on the botanical source of the starch, in water absorption capacity, molecular-weight distribution, maltooligosaccharide profile, particle-size distribution, and gel-forming properties. Ground FPS, obtained as a by-product of cold-pressed oil production, was also used in the study. The raw material was supplied by Food Processing System Monika USS, located in Targowisko near Krakow, Poland (557 Targowisko, 31-015 Targowisko). The moisture content of the FPS was 15.03%. Before analysis, the FPS was ground using a Laboratory Mill 3100 (Perten Instruments, Springfield, IL, USA) and subsequently sieved through a 0.88 mm mesh to ensure a uniform particle size.

The final material used for the analyses consisted of cookies containing above mentioned maltodextrins and FPS.

### 3.2. Methods

#### 3.2.1. Experimental Design

The experiment was designed and conducted in three stages.

Stage I—Determination of the most favorable level of margarine replacement with maltodextrin in cookies. The objective of Stage I was to compare different levels of margarine replacement with maltodextrin (5–25%) and select the variant with the most favorable physical and sensory properties. Commercially available maltodextrin with a DE of 17 (Agnex, Poland) was used. One control sample containing the full amount of margarine and five experimental variants (A1–A5) were prepared. In these variants, 5%, 10%, 15%, 20%, and 25% of the original amount of margarine, respectively, was replaced with an equivalent weight of maltodextrin. The total weight of the ingredients remained unchanged in all formulations. The following formulations were used to prepare the Stage I cookies:

All variants were prepared using the same procedure. The ingredients were weighed and mixed for 5 min in a spiral mixer (SP 12, Diosna, Osnabrück, Germany). The dough was then rolled to a thickness of 5 mm, cut into shapes, and baked at 210 °C for 12 min. After cooling, the cookies were stored in glass containers at 22 °C. Based on the results of the physical analyses, including specific volume, texture, and color, and the sensory evaluation, the variant with the most favorable properties was selected as the basis for the subsequent stage.

Stage II—Comparison of maltodextrins obtained from light-fleshed and purple-fleshed potato starch. After the most favorable margarine replacement level had been selected in Stage I, a comparative experiment was conducted in Stage II to determine whether the type of potatoes used to produce the maltodextrin affected the properties of the final cookies. Two types of maltodextrin were used: maltodextrin obtained from the starch of the light-fleshed Lord and Tajfun cultivars and maltodextrin obtained from the starch of the purple-fleshed Violetta and Blue Star cultivars. Three cookie variants were prepared: control cookies (K II), cookies containing maltodextrin obtained from light-fleshed potato starch, and cookies containing maltodextrin obtained from purple-fleshed potato starch. In both maltodextrin variants, 10% of the margarine was replaced with maltodextrin according to the formulation presented in Table 9.

Stage III—Evaluation of maltodextrin as a margarine replacer and FPS as a functional enrichment ingredient (Table 10). Based on the results of Stage I, the most favorable margarine replacement level among those tested was selected. As no substantial overall quality differences were found in Stage II between the cookies containing maltodextrin obtained from light-fleshed and purple-fleshed potato starch, both types were retained for further investigation. Ground FPS was used as a partial wheat flour replacer at levels of 10%, 20%, and 30%, calculated relative to the original wheat flour amount and replacing flour on a weight-for-weight basis. Accordingly, 9.1, 18.2, and 27.3 g of the original 91.0 g of wheat flour were replaced with FPS, respectively. The resulting cookies were analyzed for their bioactive properties, physical characteristics, sensory attributes, and consumer acceptability.

#### 3.2.2. Determination of Dry Matter Content

Empty, uncovered weighing vessels were dried in an oven at 130 °C for 1 h and then transferred to a desiccator to cool. After cooling, each empty vessel with its lid was weighed, and 1 g of the analyzed sample was placed inside it. The uncovered vessels were dried at 130 °C for 1 h and subsequently cooled in a desiccator. After cooling, each vessel was covered with its lid and weighed together with the dried sample. Dry matter content was calculated using the following equation:

$$s=\frac{\left(m2-m\right)}{\left(m1-m\right)}$$

where

*m*—mass of the empty weighing vessel with its lid [g];
*m*1—mass of the weighing vessel with its lid and the sample before drying [g];
*m*2—mass of the weighing vessel with its lid and the sample after drying [g].

#### 3.2.3. Determination of Bioactive Compounds

*Sample Preparation—Ethanolic Extraction*

For extraction, 0.6 g of the sample was mixed with 30 mL of 80% ethanol. Extraction was carried out for 2 h at room temperature (23 °C) in the dark using a shaking water bath (WB 22, Memmert GmbH + Co. KG, Schwabach, Germany). The samples were then centrifuged at 4500 rpm (1050× *g*) for 15 min using a laboratory centrifuge (MPW-350, MPW Med. Instruments, Warsaw, Poland). The resulting supernatant was separated from the precipitate and stored at −20 °C until further analysis.

*UHPLC-DAD-ESI-HRMS/MS Analysis of Phenolic Compounds*

Qualitative and quantitative analyses of phenolic compounds in 80% ethanolic extracts obtained from defatted flaxseed press cake and cookie samples were performed using ultra-high-performance liquid chromatography coupled with diode-array detection and high-resolution tandem mass spectrometry (UHPLC-DAD and UHPLC-ESI-HRMS/MS). The analytical procedure was based on the method described by Oracz et al. [93], with modifications adapted to the investigated matrices.

UHPLC-DAD analysis was carried out using a Dionex UltiMate 3000 UHPLC+ system equipped with a diode-array detector (Thermo Fisher Scientific Inc., Waltham, MA, USA). High-resolution mass-spectrometric analysis was performed using a Transcend TLX-2 multiplexed liquid chromatography system coupled to a Q-Exactive Orbitrap mass spectrometer equipped with a heated electrospray ionization interface (HESI-II; Thermo Scientific, Hudson, NH, USA).

Chromatographic separation was achieved on an Accucore C18 column (2.1 × 150 mm, 2.6 µm particle size; Thermo Scientific) maintained at 30 °C. The mobile phase consisted of 0.1% formic acid in water (eluent A) and acetonitrile (eluent B), and the flow rate was set at 0.35 mL/min. The following gradient program was applied: 0–8 min, 1–5% B; 8–15 min, 5–8% B; 15–20 min, 8–10% B; 20–25 min, 10–15% B; 25–35 min, 15–20% B; 35–40 min, 20–25% B; 40–50 min, 25–90% B; 50–53 min, 90% B; and 53–58 min, 90–1% B. The initial mobile-phase composition of 1% B was subsequently maintained for 7 min to re-equilibrate the column before the next injection. The total chromatographic run time was 65 min. Chromatograms were recorded at 280 nm for hydroxybenzoic acids and their derivatives and at 320 nm for hydroxycinnamic acids and their derivatives. The diode-array detector was also used to record the UV–Vis absorption spectra of the separated compounds.

High-resolution mass-spectrometric analysis was conducted in negative electrospray ionization mode. The spray voltage was set at 4.5 kV, the capillary temperature at 275 °C, and the auxiliary-gas heater temperature at 320 °C. Nitrogen was used as both the sheath and auxiliary gas at flow rates of 35 and 15 arbitrary units, respectively. Full-scan mass spectra were acquired over an m/z range of 100–1500. Tandem mass spectra were obtained using higher-energy collisional dissociation (HCD) at a normalized collision energy of 20%. Phenolic compounds were identified by comparing their retention times, UV–Vis absorption maxima, deprotonated molecular ions [M−H]^−^, accurate mass values, and MS^2^ fragmentation patterns with those of authentic reference standards analyzed under the same chromatographic conditions (Table S1). The identification was additionally verified using previously published mass-spectrometric data. Compounds showing agreement with the corresponding reference standards were considered positively identified. The compounds identified in the analyzed extracts included gallic acid, protocatechuic acid (3,4-dihydroxybenzoic acid), p-hydroxybenzoic acid (4-hydroxybenzoic acid), neochlorogenic acid, chlorogenic acid, p-coumaric acid, ferulic acid, and sinapic acid.

Individual phenolic compounds were quantified from the UHPLC-DAD peak areas using the external standard method. Calibration solutions of gallic, protocatechuic, p-hydroxybenzoic, neochlorogenic, chlorogenic, p-coumaric, ferulic, and sinapic acids were prepared over a concentration range of 0.01–100 mg/L. Calibration curves were constructed by plotting peak area against analyte concentration, and method linearity was evaluated using the corresponding regression equations and correlation coefficients. The limits of detection (LOD) and quantification (LOQ) were defined as the lowest analyte concentrations providing signal-to-noise ratios of at least 3 (S/N ≥ 3) and 10 (S/N ≥ 10), respectively. The content of individual phenolic compounds was expressed as milligrams per 100 g of sample (mg/100 g DM).

*Spectrophotometric Determination of Total Phenolic Content (TPC) Using the Folin–Ciocalteu Method*

Total polyphenol content (TPC) was determined spectrophotometrically using the Folin–Ciocalteu reagent according to Singleton et al. [45]. A 5 mL aliquot of the 80% ethanolic extract was diluted to a final volume of 50 mL with distilled water. Subsequently, 5 mL of the diluted extract was mixed with 0.25 mL of Folin–Ciocalteu reagent, previously diluted 1:1 (*v*/*v*) with distilled water, and 0.5 mL of 7% Na_2_CO_3_ solution. The mixture was vortexed (WF2, Janke & Kunkel, Staufen, Germany) and incubated in the dark for 30 min. Absorbance was measured at 760 nm using a Helios Gamma spectrophotometer (100–240, Runcorn, UK). The results were calculated from a catechin calibration curve, corrected for the dilution factor and sample dry matter, and expressed as mg catechin (CE)/100 g DM.

*Spectrophotometric Determination of Total Polyphenol, Phenolic Acid and Flavonols Content*

Total polyphenol, phenolic acid and flavonol content were determined spectrophotometrically according to Mazza et al. [46], as modified by Oomah et al. [47]. A 0.1 mL aliquot of the 80% ethanolic extract was transferred to a test tube and mixed with 2.4 mL of 2% HCl in 75% ethanol. The mixture was vortexed (WF2, Janke & Kunkel, Staufen, Germany), and absorbance was measured using a Helios Gamma spectrophotometer (100–240, Runcorn, UK) at 280 nm for total polyphenols, 320 nm for phenolic acids and 360 nm for flavonols. A mixture of 2% HCl in 75% ethanol and 80% ethanol was used as the blank. The results were calculated from the corresponding calibration curves and expressed as mg catechin (CE)/100 g DM for total polyphenols, mg ferulic acid (FAE)/100 g DM for phenolic acids and mg quercetin (QE)/100 g DM for flavonols.

*Determination of Flavonoid Content*

Flavonoid content was determined spectrophotometrically according to El Hariri et al. [94]. A 0.5 mL aliquot of the 80% ethanolic extract was mixed with 1.8 mL of distilled water and 0.2 mL of 2-aminoethyl diphenylborinate solution. The mixture was vortexed (WF2, Janke & Kunkel, Staufen, Germany), and absorbance was measured at 404 nm using a Helios Gamma spectrophotometer (100–240, Runcorn, UK). A blank containing 0.5 mL of 80% ethanol, 1.8 mL of distilled water, and 0.2 mL of 2-aminoethyl diphenylborinate solution was prepared in parallel. The results were calculated from a rutin calibration curve and expressed as mg rutin (RE)/100 g DM.

*Determination of DPPH Radical-Scavenging Activity*

DPPH radical-scavenging activity was determined according to Brand-Williams et al. [95]. A 1 mL aliquot of the 80% ethanolic extract was mixed with 4 mL of DPPH solution prepared by dissolving 0.012 g of 2,2-diphenyl-1-picrylhydrazyl in 100 mL of ethanol. After thorough mixing, absorbance was measured at 517 nm using a spectrophotometer. Trolox (6-hydroxy-2,5,7,8-tetramethylchromane-2-carboxylic acid) was used as the reference standard (R^2^ = 0.9829). The results were expressed as microM Trolox/kg DM.

*Determination of ABTS Radical-Scavenging Activity*

ABTS radical-scavenging activity was determined according to Re et al. [96]. An appropriately diluted aliquot of the 80% ethanolic extract was mixed with ABTS•+ solution [2,2′-azinobis(3-ethylbenzothiazoline-6-sulfonic acid)]. The mixture was vortexed (WF2, Janke & Kunkel, Staufen, Germany) and incubated in the dark for 6 min. Absorbance was then measured at 734 nm using a Helios Gamma spectrophotometer (100–240, Runcorn, UK). A blank was prepared in parallel. The results were calculated from a Trolox calibration curve and expressed as microM Trolox/kg DM.

#### 3.2.4. Physical Properties

*Specific Volume.*

After baking and cooling for 1 h at room temperature, the specific volume of the cookies was measured using a VolScan Profiler 600 laser-based volume measurement system (Stable Micro Systems, Godalming, UK). The specific volume was calculated according to the following equation: Specific volume (cm^3^/100 g) = [V (cm^3^)/m (g)] × 100 where V is the volume of the cookie measured using the VolScan Profiler 600 and m is the mass of the cookie. The results were expressed as cm^3^/100 g.

*Texture Analysis: Determination of Maximum Force and Cutting Energy*

Texture analysis was performed on the day of baking and after 30 days of storage under controlled conditions using a TA-XT2 Plus texture analyzer (Stable Micro Systems, Godalming, UK) equipped with a Warner–Bratzler blade set with a flat cutting blade. The cutting speed was 3 mm/s, and the data were analyzed using Exponent software, version 4.0.13.0. Each measurement was performed in five replicates (*n* = 5). The cookies were stored in glass jars in the dark at a constant temperature of 22 °C [31].

The following parameters were recorded:

- Hardness [N]—the maximum force required to cut the sample.
- Cutting energy [N·s]—the total energy required to cut the sample, calculated as the area under the force–time curve (Area F–T 1:2).

*Color Analysis*

The color parameters of the cookie samples were measured in reflectance mode using a CM-3500d spectrophotometer (Konica Minolta, Tokyo, Japan). Measurements were performed in the CIELAB color space using a 10° standard observer and a 30 mm aperture. Before measurement, the samples were placed in 55 mm diameter Petri dishes. The following color coordinates were recorded:

L*—lightness, ranging from 0 for black to 100 for white;
a*—the green-to-red coordinate, with negative values corresponding to green and positive values to red;
b*—the blue-to-yellow coordinate, with negative values corresponding to blue and positive values to yellow.

Color changes in the enriched cookies were evaluated relative to the control sample using the CIEDE2000 [97] total color difference parameter (Δ*E*_00_). This calculation considers corrected differences in lightness (Δ*L*′), chroma (Δ*C*′), and hue (Δ*H*′), together with interaction terms between these components. By accounting for the nonuniform response of human vision to color changes, CIEDE2000 provides an estimate of color differences that more closely corresponds to their visual perception.

$$\Delta E_{00}=\sqrt{{\left(\frac{\Delta L^{\prime }}{k_{L}S_{L}}\right)}^{2}+{\left(\frac{\Delta C^{\prime }}{k_{C}S_{C}}\right)}^{2}+{\left(\frac{\Delta H^{\prime }}{k_{H}S_{H}}\right)}^{2}+R_{T}\left(\frac{\Delta C^{\prime }}{k_{C}S_{C}}\right)\left(\frac{\Delta H^{\prime }}{k_{H}S_{H}}\right)}$$

where

Δ*L*′—difference in lightness,
Δ*C*′—difference in chroma,
Δ*H*′—difference in hue,
*S_L_*, *S_C_*, *S_H_*—weighting functions for lightness, chroma, and hue, respectively,
*R_T_*—rotation term accounting for the interaction between hue and chroma,
*k_L_*, *k_C_*, *k_H_*—parametric correction factors.

Interpretation of color difference:

– Δ*E*_00_ < 0.8—invisible difference, imperceptible to the human eye.
– Δ*E*_00_ = 0.8–1.5—very small difference, barely noticeable.
– Δ*E*_00_ = 1.5–3.0—small difference, noticeable but still acceptable.
– Δ*E*_00_ = 3.0–5.0—moderate difference, clearly visible.
– Δ*E*_00_ > 5.0—large difference, easily noticeable.

#### 3.2.5. Consumer Acceptance Evaluation

Consumer acceptance of the cookie samples was assessed under conditions conforming to the general requirements of PN-ISO 8589:1998 [98]. Six, three, and seven cookie formulations were evaluated in Stages I, II, and III, respectively (Table 11). The consumer evaluation was conducted by 14 adult participants aged 20–60 years, including seven women and seven men. Prior to the evaluation, participants underwent preliminary health screening to confirm their eligibility for sensory assessment.

The samples were coded to conceal their identity and presented to participants in a randomized order. Cookies were evaluated at room temperature and were presented as whole cookies, cross-sections, and bite-sized pieces to allow assessment of their external appearance, internal structure, and sensory characteristics. Water was provided for palate cleansing between successive samples. The evaluated attributes included external appearance, color, aroma, taste, hardness, and overall attractiveness. Each attribute was rated using a 9-point scale, with higher scores indicating greater consumer acceptance. The scores were used to determine and compare consumer acceptance among the cookie formulations within each experimental stage. The study was conducted in accordance with the ethical principles of the Declaration of Helsinki [99]. Participation was voluntary, and informed consent was obtained from all participants. Participant privacy was protected, and any potential discomfort associated with the sensory evaluation was minimized.

The participants evaluated each sample using a nine-point hedonic scale:

9—extremely desirable,
8—very desirable,
7—desirable,
6—slightly desirable,
5—neither desirable nor undesirable,
4—slightly undesirable,
3—undesirable,
2—very undesirable,
1—extremely undesirable.

#### 3.2.6. Statistical Analysis

For the HPLC analysis of individual phenolic compounds, data normality and homogeneity of variance were assessed using the Shapiro–Wilk and Levene tests, respectively. Normally distributed data with homogeneous variances were analyzed using one-way analysis of variance (ANOVA), followed by Tukey’s post hoc test. Welch’s ANOVA followed by the Games–Howell post hoc test was applied to normally distributed data with unequal variances. When the assumptions of parametric analysis were not met, the Kruskal–Wallis test followed by Dunn’s post hoc test with Bonferroni correction was used.

For the spectrophotometric analyses and physical parameters, including texture, specific volume, color, and consumer acceptance scores, differences between mean values were assessed using one-way ANOVA followed by Tukey’s post hoc test at *p* < 0.05.

In Stage III, the control sample and samples containing different levels of FPS were analyzed separately within the two maltodextrin series: maltodextrin obtained from purple-fleshed potato starch (F) and maltodextrin obtained from light-fleshed potato starch (J). Significant differences between mean values within each series were indicated by different lowercase letters. Values marked with different letters differed significantly at *p* < 0.05. Letters were assigned in ascending order of the mean values, with “a” representing the lowest statistical group.

Additionally, for the HPLC data obtained in Stage III, a two-way ANOVA was performed for the six FPS-containing variants (B1J–B3J and B1F–B3F) to evaluate the effects of FPS incorporation level (10%, 20%, and 30%), maltodextrin type (J and F), and their interaction (FPS incorporation level × maltodextrin type) on the individual phenolic acids and their total content. The K III control was excluded from the two-way ANOVA because it contained neither FPS nor maltodextrin and therefore did not represent a level within the 3 × 2 factorial design. Statistical significance was established at *p* < 0.05. Detailed results of the two-way ANOVA are presented in Supplementary Table S2.

Corresponding BXF and BXJ samples containing the same FPS incorporation level were also compared pairwise (B1F vs. B1J, B2F vs. B2J, and B3F vs. B3J). Depending on data distribution and variance homogeneity, these comparisons were performed using Student’s t-test, Welch’s t-test, or the Mann–Whitney U test. Statistical significance was denoted as follows: *p* < 0.05, *p* < 0.01, and *p* < 0.001; ns indicates a nonsignificant difference.

Pearson’s correlation coefficients (r) were calculated to assess the relationships between phenolic compound contents and the antioxidant activity of the cookies. To provide an integrated graphical representation of the HPLC results, log_2_ fold-change values were calculated relative to the control mean according to the following equation: log_2_ fold change = log_2_ (sample mean/control mean). Positive values indicate an increase relative to the control, whereas negative values indicate a decrease.

## 4. Conclusions

- Replacing 10% of the margarine with maltodextrin with a dextrose equivalent of 17 provided the most favorable overall quality profile. This variant had approximately 18% lower hardness and only 3% lower specific volume than the control and received the highest sensory score among the partially fat-replaced samples.
- The incorporation of FPS produced a dose-dependent increase in bioactive compound content. Total polyphenol content determined using the Folin–Ciocalteu method increased from 31.47 to a maximum of 424.11 mg catechin/100 g DM, representing an approximately 13.5-fold increase, while flavonoid content increased approximately 3.4 times.
- Enrichment also increased the antioxidant activity of the cookies. The highest values were 64.17 microM Trolox/kg DM in the ABTS assay and 20.65 microM Trolox/kg DM in the DPPH assay, corresponding to approximately 4.6- and 4.9-fold increases, respectively, compared with the control.
- HPLC analysis identified eight phenolic acids. Their total content increased from 2.90 mg/100 g DM in the control to 17.01 mg/100 g in B3J and 19.11 mg/100 g DM in B3F. Gallic acid was the predominant phenolic acid in the enriched samples, reaching 12.47–13.82 mg/100 g DM. Variants containing maltodextrin obtained from purple-fleshed potato starch generally had higher contents of the identified phenolic compounds. Two-way ANOVA confirmed significant effects of both FPS incorporation level and maltodextrin type on the total content of identified phenolic acids, as well as a significant interaction between these factors (*p* < 0.001).
- Increasing the proportion of FPS to 20–30% increased cookie hardness, resulted in a darker color, and reduced sensory acceptability. The most favorable balance between bioactive compound content and product quality was obtained for B1J and B1F, which contained 10% FPS. Among these variants, B1F had a higher phenolic compound content.
- The findings indicate that the valorization of FPS, a by-product of the oil industry, may support the development of partially fat-replaced cookies characterized by increased phenolic compound content and enhanced in vitro antioxidant capacity. These results relate to the compositional characteristics of the products and their antioxidant properties determined in vitro and should not be directly interpreted as evidence of health-promoting effects. The assessment of potential biological and health-promoting effects should therefore be the subject of further research. The developed cookies may provide an alternative to conventional confectionery products while contributing to the principles of the circular economy.

## Figures and Tables

**Table 1:** Hardness and cutting energy of the cookie samples analyzed in Stage I.

Sample	Hardness [N]	Cutting Energy [N·s]
K I *	55.73 ± 1.19 ^c^**	16.78 ± 7.21 ^a^
A1_5%	39.03 ± 0.76 ^a^	24.93 ± 7.05 ^a^
A2_10%	45.94 ± 1.11 ^b^	27.26 ± 1.81 ^b^
A3_15%	55.31 ± 0.69 ^c^	30.29 ± 0.63 ^b^
A4_20%	61.02 ± 5.71 ^c^	31.81 ± 6.12 ^b^
A5_25%	67.24 ± 6.51 ^d^	32.97 ± 6.29 ^b^

*—K I—control sample containing the full amount of margarine and no maltodextrin; A1_5%—sample in which 5% of the margarine was replaced with maltodextrin; A2_10%—sample in which 10% of the margarine was replaced with maltodextrin; A3_15%—sample in which 15% of the margarine was replaced with maltodextrin; A4_20%—sample in which 20% of the margarine was replaced with maltodextrin; A5_25%—sample in which 25% of the margarine was replaced with maltodextrin. **—values are presented as mean ± SD (*n* = 5), different letters within the same column indicate statistically significant differences according to Tukey’s test (*p* < 0.05).

**Table 2:** Hardness and cutting energy of the cookie samples analyzed in Stage II.

Sample	Hardness [N]	Cutting Energy [N·s]
K II	45.66 ± 4.84 ^b^*	21.68 ± 3.17 ^a^
MJ	36.14 ± 5.73 ^a^	20.37 ± 2.13 ^a^
MF	32.19 ± 6.15 ^a^	26.07 ± 3.74 ^b^

*—values are presented as mean ± SD (*n* = 5). Different letters within the same column indicate statistically significant differences according to Tukey’s test (*p* < 0.05).

**Table 3:** Contents of selected groups of polyphenolic compounds in cookies.

Sample	TPC Using the F–C Reagent (mg Catechin/100 g DM)	TPC Without the F–C Reagent (mg Catechin/100 g DM)	Flavonoids (mg Rutin/100 g DM)	Flavonols (mg Quercetin/100 g DM)	Phenolic Acids (mg Ferulic Acid/100 g DM)
K III	31.47 ± 1.99 ^a^*	27.91 ± 0.63 ^a^	15.65 ± 0.00 ^a^	nd **	nd
B1J	170.79 ± 3.99 ^b^	74.59 ± 1.67 ^b^	31.29 ± 1.79 ^b^	nd	7.08 ± 0.27 ^a^
B2J	219.15 ± 3.99 ^d^	93.55 ± 1.26 ^c^	39.32 ± 4.78 ^c^	nd	10.43 ± 0.27 ^c^
B3J	416.04 ± 3.99 ^f^	165.4 ± 1.67 ^f^	53.68 ± 1.20 ^d^	2.77 ± 0.33 ^b^	21.89 ± 0.27 ^e^
B1F	201.88 ± 1.99 ^c^	104.51 ± 1.26 ^d^	32.55 ± 4.78 ^b^	nd	8.83 ± 0.48 ^b^
B2F	315.87 ± 3.99 ^e^	124.55 ± 2.19 ^e^	41.85 ± 2.39 ^c^	0.69 ± 0.00 ^a^	14.25 ± 0.28 ^d^
B3F	424.11 ± 6.91 ^f^	165.77 ± 0.63 ^f^	53.68 ± 1.47 ^d^	2.77 ± 0.32 ^b^	24.6 ± 0.48 ^f^

*—different letters within the same column indicate statistically significant differences according to Tukey’s test (*p* < 0.05). Values are presented as mean ± standard deviation (*n* = 4). **—not detected.

**Table 4:** Phenolic compound content in samples containing purple maltodextrin [mg/100 g DM].

Compound	K III	B1F	B2F	B3F
Gallic acid	1.62 ± 0.13 ^a^*	9.47 ± 0.49 ^b^	11.54 ± 0.61 ^c^	12.47 ± 0.66 ^c^
3,4-Dihydroxybenzoic acid	0.08 ± 0.11 ^a^	0.68 ± 0.13 ^b^	1.35 ± 0.17 ^c^	1.57 ± 0.18 ^c^
4-Hydroxybenzoic acid	0.08 ± 0.11 ^a^	0.44 ± 0.11 ^b^	0.08 ± 0.11 ^a^	0.08 ± 0.11 ^a^
Neochlorogenic acid	0.08 ± 0.11 ^a^	0.58 ± 0.12 ^b^	1.83 ± 0.14 ^c^	1.98 ± 0.14 ^c^
Chlorogenic acid	0.49 ± 0.13 ^a^	2.09 ± 0.15 ^b^	2.14 ± 0.15 ^b^	1.97 ± 0.14 ^b^
p-Coumaric acid	0.28 ± 0.11 ^a^	0.42 ± 0.12 ^ab^	0.49 ± 0.11 ^ab^	0.56 ± 0.12 ^b^
Ferulic acid	0.04 ± 0.06 ^a^	0.31 ± 0.06 ^b^	0.40 ± 0.06 ^bc^	0.45 ± 0.06 ^c^
Sinapic acid	0.23 ± 0.06 ^b^	0.04 ± 0.06 ^a^	0.04 ± 0.06 ^a^	0.04 ± 0.06 ^a^
Sum	2.90 ^a^	14.02 ^b^	17.87 ^c^	19.11 ^c^

*—Values are expressed as mean ± standard deviation (*n* = 4). Different lowercase letters in the same row indicate statistically significant differences among the control and samples within a given maltodextrin variant (Tukey’s test, *p* < 0.05). Lettering is assigned according to increasing mean values; therefore, the lowest statistical group is marked with ‘a’. The same control sample was used as a reference for both maltodextrin variants.

**Table 5:** Phenolic compound content in samples containing light maltodextrin [mg/100 g DM].

Compound	K III	B1J	B2J	B3J
Gallic acid	1.62 ± 0.13 ^a^*	3.68 ± 0.21 ^b^	6.10 ± 0.32 ^c^	13.82 ± 0.74 ^d^
3,4-Dihydroxybenzoic acid	0.08 ± 0.11 ^a^	0.44 ± 0.12 ^b^	0.08 ± 0.11 ^a^	0.08 ± 0.11 ^a^
4-Hydroxybenzoic acid	0.08 ± 0.11 ^a^	0.08 ± 0.11 ^a^	0.08 ± 0.11 ^a^	0.08 ± 0.11 ^a^
Neochlorogenic acid	0.08 ± 0.11 ^a^	0.90 ± 0.12 ^bc^	0.72 ± 0.12 ^b^	1.07 ± 0.12 ^c^
Chlorogenic acid	0.49 ± 0.13 ^c^	1.07 ± 0.12 ^ab^	1.31 ± 0.13 ^bc^	0.96 ± 0.12 ^a^
p-Coumaric acid	0.28 ± 0.11 ^ab^	0.08 ± 0.11 ^a^	0.08 ± 0.11 ^a^	0.51 ± 0.11 ^b^
Ferulic acid-	0.04 ± 0.06 ^a^	0.04 ± 0.06 ^a^	0.04 ± 0.06 ^a^	0.40 ± 0.06 ^b^
Sinapic acid	0.23 ± 0.06 ^b^	0.24 ± 0.06 ^b^	0.04 ± 0.06 ^a^	0.09 ± 0.06 ^a^
Total	2.90 ^a^	6.53 ^ab^	8.45 ^b^	17.01 ^c^

*—Values are expressed as mean ± standard deviation (*n* = 4). Different lowercase letters in the same row indicate statistically significant differences among the control (K III) and samples within a given maltodextrin variant (Tukey’s test, *p* < 0.05). Lettering is assigned according to increasing mean values; therefore, the lowest statistical group is marked with ‘a’. The same control sample was used as a reference for both maltodextrin variants.

**Table 6:** Effect of maltodextrin type on the content of selected phenolic compounds in cookies.

Compound	B1F vs. B1J	B2F vs. B2J	B3F vs. B3J
Gallic acid	B1F > B1J ^x^***	B2F > B2J ***	B3J > B3F *
3,4-Dihydroxybenzoic acid	B1F > B1J *	B2F > B2J ***	B3F > B3J ***
4-Hydroxybenzoic acid	B1F > B1J **	n.s.	n.s.
Neochlorogenic acid	B1J > B1F **	B2F > B2J ***	B3F > B3J ***
Chlorogenic acid	B1F > B1J ***	B2F > B2J ***	B3F > B3J ***
p-Coumaric acid	B1F > B1J **	B2F > B2J **	n.s.
Ferulic acid	B1F > B1J ***	B2F > B2J ***	n.s.
Sinapic acid	B1J > B1F **	n.s.	n.s.
Total	B1F > B1J ***	B2F > B2J ***	B3F > B3J *

^x^—Asterisks indicate statistically significant differences between purple and light maltodextrin at the same incorporation level: * *p* < 0.05, ** *p* ≤ 0.01, *** *p* < 0.001; n.s.—not significant. Cell shading was used to facilitate interpretation: light purple indicates significantly higher values for samples containing purple maltodextrin (F > J), light cream indicates significantly higher values for samples containing light maltodextrin (J > F), and light gray indicates no significant differences.

**Table 7:** Heatmap of log_2_ fold-change values in contents of individual phenolic compounds relative to the control mean.

Compound	B1F	B2F	B3F	B1J	B2J	B3J
Gallic acid	2.549 *	2.835	2.946	1.187	1.916	3.095
3,4-Dihydroxybenzoic acid	3.087	4.079	4.292	2.459	0.000	0.000
4-Hydroxybenzoic acid	2.459	0.000	0.000	0.000	0.000	0.000
Neochlorogenic acid	2.870	4.516	4.629	3.492	3.170	3.748
Chlorogenic acid	2.090	2.133	2.010	−0.478	−0.186	−0.630
p-Coumaric acid	0.576	0.807	1.013	−1.807	−1.807	0.865
Ferulic acid	2.966	3.322	3.492	0.000	0.000	3.322
Sinapic acid	−2.524	−2.524	−2.524	0.061	−2.524	−1.354
Total	2.274	2.623	2.721	1.005	1.387	2.499

*—Heatmap showing log_2_ fold-change values in the contents of individual phenolic compounds in samples containing maltodextrin derived from starch isolated from purple-fleshed potatoes (B1F–B3F) and maltodextrin derived from starch isolated from light-fleshed potatoes (B1J–B3J), calculated relative to the control mean. Positive values indicate an increase compared with the control, whereas negative values indicate a decrease. Values close to zero indicate little or no change relative to the control.

**Table 8:** Hardness and cutting energy of the cookie samples analyzed in Stage III on the day of baking and after 30 days of storage.

Sample	Hardness [N]	Cutting Energy [N·s]	Hardness [N]	Cutting Energy [N·s]
	Day of Baking		After 30 Days of Storage	
K III	25.24 ± 3.20 ^a^*	23.39 ± 6.72 ^a^	21.76 ± 6.90 ^a^	17.35 ± 2.42 ^a^
B1J	39.09 ± 3.96 ^bc^	27.67 ± 2.19 ^a^	46.95 ± 14.50 ^b^	36.34 ± 14.03 ^ab^
B2J	78.39 ± 1.80 ^f^	31.90 ± 8.41 ^ab^	57.90 ± 15.16 ^c^	20.03 ± 5.07 ^a^
B3J	56.82 ± 6.15 ^d^	31.19 ± 7.81 ^ab^	67.51 ± 17.11 ^d^	23.91 ± 7.42 ^a^
B1F	36.88 ± 6.51 ^b^	24.58 ± 7.43 ^a^	51.16 ± 8.12 ^bc^	28.13 ± 16.15 ^ab^
B2F	52.49 ± 6.15 ^d^	23.39 ± 7.81 ^a^	62.78 ± 17.68 ^cd^	32.80 ± 12.99 ^ab^
B3F	65.87 ± 3.98 ^e^	28.14 ± 5.62 ^a^	75.84 ± 31.45 ^d^	30.11 ± 5.94 ^ab^

*—different letters above the columns indicate statistically significant differences according to Tukey’s test (*p* < 0.05). Values are presented as mean ± standard deviation (*n* = 5).

**Table 9:** Formulation of the cookies prepared in Stage I.

Ingredient	K I	A1_5%	A2_10%	A3_15%	A4_20%	A5_25%
Wheat flour (pastry type)	182.0 g	182.0 g	182.0 g	182.0 g	182.0 g	182.0 g
Powdered sugar	23.0 g	23.0 g	23.0 g	23.0 g	23.0 g	23.0 g
Margarine	104.0 g	98.8 g	93.6 g	88.4 g	83.2 g	78.0 g
Egg mixture	16.0 g	16.0 g	16.0 g	16.0 g	16.0 g	16.0 g
Maltodextrin	-	5.2 g	10.4 g	15.6 g	20.8 g	26.0 g

**Table 10:** Formulation of the cookies prepared in Stage III.

Ingredient [g]	K III	B1 J	B1 F	B2 J	B2 F	B3 J	B3 F
Wheat flour (pastry type)	91.0	81.9	81.9	72.8	72.8	63.7	63.7
Powdered sugar	11.5	11.5	11.5	11.5	11.5	11.5	11.5
Margarine	52.0	46.8	46.8	46.8	46.8	46.8	46.8
Egg mixture	8.0	8.0	8.0	8.0	8.0	8.0	8.0
Maltodextrin	-	5.2	5.2	5.2	5.2	5.2	5.2
Flaxseed press cake *	-	9.1	9.1	18.2	18.2	27.3	27.3

*—FPS replaced 10%, 20%, or 30% of the original wheat flour amount on a weight-for-weight basis.

**Table 11:** Consumer evaluation sheet for cookies.

Quality Attribute	1 Point	2 Point	3 Point	4 Point	5 Point	6 Point	7 Point	8 Point	9 Point
External appearance									
Color									
Aroma									
Taste									
Firmness									
Overall attractiveness									

## Data Availability

The data presented in this study are available upon request from the corresponding author.

## Supplementary Materials

The following supporting information can be downloaded at: https://www.mdpi.com/article/10.3390/molecules31203752/s1, Table S1. Identification of phenolic compounds in control and flaxseed-press cake-enriched partially fat-replaced cookies by UHPLC-DAD and UHPLC-ESI-HRMS/MS. Table S2. Two-way ANOVA of the effects of FPS addition level, maltodextrin type, and their interaction on the bioactive compound content and textural properties of Stage III cookies.

## Abbreviations

The following abbreviations are used in this manuscript:

K I	Control sample in Stage I, without maltodextrin
A1_5%	Sample in which 5% of the margarine was replaced with maltodextrin
A2_10%	Sample in which 10% of the margarine was replaced with maltodextrin
A3_15%	Sample in which 15% of the margarine was replaced with maltodextrin
A4_20%	Sample in which 20% of the margarine was replaced with maltodextrin
A5_25%	Sample in which 25% of the margarine was replaced with maltodextrin
K II	Control sample in Stage II, without maltodextrin
MJ	Sample containing maltodextrin obtained from light-fleshed potato starch
MF	Sample containing maltodextrin obtained from purple-fleshed potato starch
K III	Control sample in Stage III, without flaxseed press cake or maltodextrin
B1J	Sample containing 10% flaxseed press cake and maltodextrin obtained from light-fleshed potato starch
B1F	Sample containing 10% flaxseed press cake and maltodextrin obtained from purple-fleshed potato starch
B2J	Sample containing 20% flaxseed press cake and maltodextrin obtained from light-fleshed potato starch
B2F	Sample containing 20% flaxseed press cake and maltodextrin obtained from purple-fleshed potato starch
B3J	Sample containing 30% flaxseed press cake and maltodextrin obtained from light-fleshed potato starch
B3F	Sample containing 30% flaxseed press cake and maltodextrin obtained from purple-fleshed potato starch
DE	Dextrose equivalent—a measure of the degree of maltodextrin hydrolysis
SDG	Secoisolariciresinol diglucoside—the predominant lignan in flaxseed
F–C	Folin–Ciocalteu reagent
TPC	Total polyphenol content
ABTS	2,2′-Azinobis(3-ethylbenzothiazoline-6-sulfonic acid)—a compound used to determine antioxidant activity
DPPH	2,2-Diphenyl-1-picrylhydrazyl—a compound used to determine antioxidant activity
HPLC	High-performance liquid chromatography
UHPLC	Ultra-high-performance liquid chromatography
DAD	Diode-array detector
ESI-MS/MS	Electrospray ionization tandem mass spectrometry
HESI-II	Heated electrospray ionization II
GAE	Gallic acid
CE	Catechin
RE	Rutin
QE	Quercetin
TX	Trolox
ANOVA	Analysis of variance
SD	Standard deviation
UV–Vis	Ultraviolet–visible spectroscopy
FFA	Free fatty acids
CIELAB	Color space defined by the International Commission on Illumination
L*	Lightness coordinate in the CIELAB color space
a*	Color coordinate ranging from green (−) to red (+)
b*	Color coordinate ranging from blue (−) to yellow (+)
Δ*E*_00_	Total color difference calculated using the CIEDE2000 formula
RSA	Radical-scavenging activity
ISO	International Organization for Standardization
