# Supplementary material for: Effect of Flaxseed Press Cake (Linum usitatissimum L.) Incorporation on the Polyphenol Content, Antioxidant Potential, and Quality of Partially Fat-Replaced Cookies
Source: Molecules. 2026 Oct 19;31(20):3752. doi: 10.3390/molecules31203752 (PMC13707727; doi:10.3390/molecules31203752)
Supplement: Supplementary file 1 [file molecules-31-03752-s001.zip › molecules-4587873-supplementary.pdf]

Table S1. Identification of phenolic compounds in control and flaxseed-press cake-enriched partially fat-replaced cookies by UHPLC-DAD and UHPLC-ESI-HRMS/MS.

Peak no.	RT (min)	Identified compounds	UV data (λ_{max}, nm)	$[M-H]^-$ (*m/z*)	MS^2 fragment ions (m/z)
1	2.93	Gallic acid	270.3	169.0	125.0, 97.0
2	5.25	3,4-Dihydroxybenzoic acid	259.1, 293.1	153.0	109.0
3	8.46	4-Hydroxybenzoic acid	191.6, 254.9	137.0	93.0
4	8.95	Neochlorogenic acid	324.1	353.1	191.1
5	13.36	Chlorogenic acid	324.3	353.1	191.1
6	18.36	*p*-Coumaric acid	309.2	163.1	119.0
7	22.31	Ferulic acid	322.4	193.0	134.0
8	24.21	Sinapic acid	323.7	223.0	179.1

RT - retention time; λ_{max} - maximum absorbance.

Table S2. Two-way ANOVA of the effects of FPS addition level, maltodextrin type, and their interaction on the bioactive compound content and textural properties of Stage III cookies.

HPLC compound	FPS level F (*p*)	Maltodextrin type F (*p*)	FPS × MD F (*p*)
Gallic acid	309.14 (<0.001)	225.05 (<0.001)	112.26 (<0.001)
3,4-Dihydroxybenzoic acid	7.14 (0.005)	304.61 (<0.001)	45.16 (<0.001)
4-Hydroxybenzoic acid	6.73 (0.007)	6.73 (0.018)	6.73 (0.007)
Neochlorogenic acid	78.82 (<0.001)	118.20 (<0.001)	72.82 (<0.001)
Chlorogenic acid	7.20 (0.005)	290.46 (<0.001)	1.19 (0.327)
p-Coumaric acid	15.03 (<0.001)	32.61 (<0.001)	5.34 (0.015)
Ferulic acid	42.56 (<0.001)	93.67 (<0.001)	15.41 (<0.001)
Sinapic acid	6.77 (0.006)	13.02 (0.002)	6.77 (0.006)
Total identified phenolic acids	104.42 (<0.001)	203.01 (<0.001)	24.22 (<0.001)
